# Taboos and Self-Censorship Among U.S. Psychology Professors

**DOI:** 10.1177/17456916241252085

**Published:** 2024-05-16

**Authors:** Cory J. Clark, Matias Fjeldmark, Louise Lu, Roy F. Baumeister, Stephen Ceci, Komi Frey, Geoffrey Miller, Wilfred Reilly, Dianne Tice, William von Hippel, Wendy M. Williams, Bo M. Winegard, Philip E. Tetlock

**Affiliations:** 1 School of Arts and Sciences, The Wharton School, University of Pennsylvania; 2 Independent; 3 Stanford Business School, Stanford University; 4 School of Psychology, University of Queensland; 5 Department of Psychology, Cornell University; 6 Research, Foundation for Individual Rights and Expression; 7 Department of Psychology, University of New Mexico; 8 Political Science Program, School of Criminal Justice and Government Relations, Kentucky State University; 9 Department of Psychology, Brigham Young University; 10 Research with Impact; 11 Department of Psychology, Cornell University; 12 School of Arts and Sciences, The Wharton School, University of Pennsylvania

**Keywords:** taboos, metascience, self-censorship, academic freedom, psychology, organizational behavior, conflict

## Abstract

We identify points of conflict and consensus regarding (a) controversial empirical claims and (b) normative preferences for how controversial scholarship—and scholars—should be treated. In 2021, we conducted qualitative interviews (*n* = 41) to generate a quantitative survey (*N* = 470) of U.S. psychology professors’ beliefs and values. Professors strongly disagreed on the truth status of 10 candidate taboo conclusions: For each conclusion, some professors reported 100% certainty in its veracity and others 100% certainty in its falsehood. Professors more confident in the truth of the taboo conclusions reported more self-censorship, a pattern that could bias perceived scientific consensus regarding the inaccuracy of controversial conclusions. Almost all professors worried about social sanctions if they were to express their own empirical beliefs. Tenured professors reported as much self-censorship and as much fear of consequences as untenured professors, including fear of getting fired. Most professors opposed suppressing scholarship and punishing peers on the basis of moral concerns about research conclusions and reported contempt for peers who petition to retract papers on moral grounds. Younger, more left-leaning, and female faculty were generally more opposed to controversial scholarship. These results do not resolve empirical or normative disagreements among psychology professors, but they may provide an empirical context for their discussion.

From Galileo’s clashes with his Aristotelian peers to the daily dramas on X (formerly Twitter), conflict is inevitable in science. Although sometimes vexing, conflict and competition drive scientific progress by motivating scholars to forward their most persuasive arguments and data. But conflicts can also delay scientific progress by creating peer hostility, setting boundaries on what is thinkable, and intimidating scholars into conformity or silence (Clark et al., 2024; Haidt, 2020; Mandel & Tetlock, 2016). Among a sample of U.S. psychology professors, we identified a set of controversial research conclusions, and we explored the professors’ empirical beliefs, self-censorship, desire to discourage controversial research, support for punishing peers who put forward controversial conclusions, and normative preferences surrounding academic freedom. We found several sources of conflict, including large disagreements regarding the empirical accuracy of 10 controversial research conclusions. But we also identified sources of agreement, including a popular normative view that harm concerns are not a legitimate reason to suppress research. We make no claims regarding the accuracy of controversial empirical conclusions, nor do we make claims regarding the optimal norms and policies for science. We seek only to illuminate popular opinions where they exist and disagreements where they do not.

## Scientific Conflict

Scientific claims often directly contradict other scientific claims (Clark & Tetlock, 2022). Many scientists, public intellectuals, and philosophers of science believe that disagreement and viewpoint diversity fuel scientific progress by motivating competing scholars to scrutinize and identify flaws in opponents’ claims and to collect more and better data (Ceci & Williams, 2022; Duarte et al., 2015; Lamers et al., 2021; Rauch, 2021; Sarewitz, 2011). Although scientific disagreement can be constructive, it can also be detrimental. For example, during the late 1800s, paleontologists Cope and Marsh, in competition to discover the most fossils, resorted to various unprofessional and counterproductive tactics—even damaging fossils and digging sites—to undermine and humiliate each other (Thomson, 2008). But this rivalry also sparked a surge of scientific progress and became known as both the Great Dinosaur Rush and the Bone Wars.

Scientific conflict can impede and catalyze scientific progress, but it is rarely studied empirically. Published and public debates among scientists illuminate existing scientific disagreements, but they are not inclusive and thus do not reveal the full distribution of perspectives. Scholars can learn about ongoing and emerging debates in journals that publish replies and commentaries, but these cases of scientific disputation are curated for particular perspectives and include only a small number of scholars. These public exchanges likely systematically underrepresent existing but socially costly scientific perspectives, such as views that contradict widely shared progressive values (Honeycutt & Freberg, 2017). In the present work, we administered an anonymous online survey to reduce (although probably not eliminate) socially desirable responding and more thoroughly document psychologists’ beliefs and perspectives.

Many scientific conflicts extend beyond precise and technical empirical disagreements to broader metascience issues. Indeed, some of the most significant conflicts in science concern the norms, practices, and policies that govern the institution of science. Different scholars, including university leadership, journal editorial boards, professional society leadership, high-status members of the community, and up-and-comers have different ideas about which norms and policies best improve science and society. For example, many scholars have advocated for greater transparency and reproducibility in science (e.g., Kidwell et al., 2016; Nosek et al., 2015, 2022), but not all scholars have responded favorably to these norm changes (e.g., Bahlai et al., 2019; Whitaker & Guest, 2020). In the present work, we explore a growing conflict: a perceived tension between academic freedom and morally responsible science.

In the last few years, journals such as *Nature Human Behaviour* and *Nature Communications* have either changed their publication guidelines or published editorials indicating that scientific papers may be rejected or retracted on the basis of harm concerns surrounding research conclusions (Clark, Jussim, et al., 2023; Nature Human Behaviour Editorial, 2022). Whereas university ethics boards have long protected research participants from harm, these new policies seek to protect society from putative harms that may attend the dissemination of science. Students, the public, and peer scholars increasingly target behavioral scientists for their scholarship—often because the conclusions appear harmful (Clark, Jussim, et al., 2023; German & Stevens, 2022). Social media has amplified academic controversies, with lay and academic users sometimes initiating retraction petitions, which may partially explain increasing retraction rates (Retraction Watch, 2021). Many retractions are based on legitimate evidence of data fraud or mistakes that cast serious doubt on reported conclusions (e.g., *Journal of Personality and Social Psychology*, 2023). But in some cases, moral concerns about the research conclusions themselves (e.g., that they could cause the spread of negative stereotypes) appear to have tripped alarms (AlShebli et al., 2020; Nature Communications Editorial, 2020; Savolainen, 2023). These harm concerns are now treated, at least in some cases, as legitimate reasons to suppress scholarship (Nature Human Behaviour Editorial, 2022).

Simultaneously, many scholars and organizations have sounded a different alarm about threats to academic freedom and growing censoriousness on college campuses, often stemming from harm concerns about vulnerable groups (e.g., Clark, Jussim, et al., 2023; German & Stevens, 2022; Honeycutt et al., 2023; Lukianoff & Haidt, 2019; Shibley, 2018). For example, a report titled *Scholars Under Fire* by the Foundation for Individual Rights and Expression reported hundreds of incidents of scholars targeted for their teaching or scholarship and often for speech perceived as harmful (German & Stevens, 2022). And a report called *Academic Freedom in Crisis* by the Center for the Study Partisanship and Ideology found that many academics in the United States, United Kingdom, and Canada supported dismissal campaigns for scholars who forward empirical conclusions that could harm vulnerable groups (e.g., claims such as “a higher share of women and ethnic minorities in organizations correlates with reduced organizational performance” [Kaufmann, 2021]). Whereas some scholars explicitly appeal to potential harms to criticize and suppress scholarship, other scholars consider these actions illegitimate censorship.

The precise harms presumed to conflict with academic freedom change over time—corrupting the youth (e.g., May, 2000; Ramsey & Varley, 1951), impiety (e.g., May, 2000), heresy (e.g., Numbers, 2009), threats to human dignity (Nature Human Behaviour Editorial, 2022)—but the broader conflict between empirical assertions and community values is as old as philosophy itself. A primary goal of science is to pursue an empirically accurate description of the natural world, and nature does not always conform to human social values and desires. Indeed, the claim that humans strive to climb status hierarchies (as among scientists) is likely true (Clark & Winegard, 2020; Storr, 2021), but it is not a particularly flattering view of human nature. A scholar speaking the truth—or at least what he or she sincerely believes to be true, on the basis of the evidence—may occasionally offend some or even most people. Pursuit of truth in the human behavioral sciences may be especially likely to spark moral outrage because the subjects—humans—are also the consumer. This might explain why academics in the social sciences and humanities are both more censorious and more censored than science, technology, engineering, and mathematics (STEM) scholars (German & Stevens, 2022; Kaufmann, 2021). Scientists who stumble upon undesirable truths can suppress their findings, for example, by file-drawering data (Rosenthal, 1979); if they do not, they risk becoming a victim of a “shoot the messenger” reaction from colleagues. Different scholars will calculate such risks differently. And different scholars will differentially judge peers for their risk calculations and even their peer judgments. The phrase “guilt by association” implies that affiliation with a morally suspect peer can itself be perceived as a moral violation.

The current range of beliefs and normative preferences regarding these scientific issues among psychology professors is unknown. This nescience may create pluralistic ignorance (Prentice & Miller, 1996) and preference falsification (Kuran, 1997), leading scholars to underestimate the popularity of their own views and to self-censor or misrepresent their views for fear of social sanction. In interviews and a quantitative survey, we document the range of perspectives among U.S. psychology professors surrounding issues of sociopolitically controversial scholarship, academic freedom, and the raison d’être of psychological science. Reports from similar populations often find demographic differences in related values, with younger, more left-leaning, and female faculty relatively more punitive of controversial scholarship and less supportive of academic freedom (Honeycutt et al., 2023; Kaufmann, 2021). For this reason, we explore these demographic differences as well. By allowing professors to share their views confidentially and anonymously, we hope to initiate a conversation that is more inclusive of the full diversity of perspectives. Our survey focuses both on empirical disagreement as well as normative disagreement. Although conflict regarding the optimal norms of science often stem from value conflicts that cannot be adjudicated with data, many normative disagreements rest on empirical assumptions. Here, we identify points of disagreement and consensus that are intended to provide context to current conflicts in academic psychology.

## Pilot Study

In early 2021, 41 scholars with PhDs in psychology or related disciplines were interviewed over Zoom for purposes of identifying taboo research conclusions and gathering other information for the main study. All questions in the main survey were informed by responses in the pilot study to ensure we were asking pertinent questions and providing appropriate response options. (See the Supplemental Material available online for full details.)

Overwhelmingly, the most taboo conclusions involved genetic, evolutionary, biological, or otherwise natural explanations for group differences in socially important outcomes (e.g., intelligence, education and career outcomes, socioeconomic status, criminal behavior), particularly in domains in which women underperform relative to men or Black people underperform relative to White people. But respondents mentioned a variety of taboo conclusions. We considered both popularity and diversity of responses to generate 10 distinct taboo conclusions. Respondents described conceptually similar taboo conclusions in sundry ways. To phrase each taboo conclusion in informative ways, we consulted with relevant and diverse experts. However, taboo conclusions tend to be complex and difficult to fully capture with a limited number of quantitative questions, so the phrasing is inevitably imperfect. Nonetheless, the responses to the main study corresponded very well to the pilot study, in which professors were free to formulate their responses in their own words.

## Main Study

### Method

#### Open science statement

This study was not preregistered, and our author team had no consensual hypotheses. The Qualtrics survey with verbatim study materials is included in the Supplemental Material. To encourage honest responding, we assured participants that all demographic variables and open-ended responses would be removed from the data file before we shared it publicly (to ensure anonymity). With these variables removed, the remaining portion of our data set (quantitative responses to the primary survey questions) is available on our Open Science Framework (OSF) page. Because many analyses (particularly those regarding age, gender, and ideology) are not reproducible with this limited data set, we have uploaded output pdfs of all primary analyses that included demographic variables to our OSF page.

#### Participants

In summer 2021, we collected the top 100 universities and the top 100 psychology graduate programs in the United States according to *U.S. News & World Report* rankings. After accounting for overlap and excluding two universities that either did not have psychology faculty or a psychology faculty webpage, 133 universities were included (see the Supplemental Material for a list). We collected email addresses from faculty webpages for each university. In late 2021, we invited 4,603 psychology faculty to participate, of whom 470 provided responses on at least some questions. Some professors opted to participate in a selection of questions but not all questions. For maximum inclusion, these participants were retained, and any missing data were simply allowed to remain missing. Between 415 and 419 participants completed demographic questions. Of these, participants were 57.1% male, 39.8% female, 0.5% nonbinary, and 2.6% undisclosed; 10.4% were ages 26 to 35, 30.4% ages 36 to 45, 27.5% ages 46 to 55, 17.1% ages 56 to 65, 12.3% ages 66 to 75, and 2.4% ages 76 and above. On political ideology participants leaned left (*M* = 25.04, *SD* = 18.00), with 92.6% identifying at the midpoint (50) or to the left of it. To incentivize participation, at the conclusion of the study, we gave participants the option to be redirected to a new survey where they could enter a drawing for one of 100 Amazon gift cards ($100 value).

The Supplemental Material reports various representativeness checks comparing our sample to a complete coding of 300 randomly selected members of our population as well as to four other similar samples (Buss & von Hippel, 2018; Causadias et al., 2018; Inbar & Lammers, 2012; von Hippel & Buss, 2017)^1^ along the dimensions of academic position, age, gender, and political ideology. These representativeness checks show that our sample was very similar to our population across these four variables, and observed differences never reached minimum thresholds for small effects (Cohen, 1977). To the extent that we were able to estimate potential deviations from representativeness, the following groups may have been slightly underrepresented: professors age 76 and above, females (although males were not clearly overrepresented), professors who were further politically left in general, and professors who were further politically right on economic issues. Our sample seemed closely representative along dimensions of academic position and social political ideology. However, because our survey included topics on which many professors self-censor, responses to our survey were almost certainly not perfectly representative of our population. We discuss this issue further in the General Discussion.

### Procedure

Participants were told they would be responding to 10 taboo conclusions in the social sciences that were nominated by their peers in earlier interviews. First, participants responded to three questions regarding each conclusion on 101-point sliding scales (ranging from 0 to 100): “How confident are you in the truth or falsity of this statement?” (responses ranged from *100% confident it is false* to *100% confident it is true*), “If the topic came up in a professional setting—for example, at a conference—how reluctant would you feel about sharing your beliefs on this topic openly?” (responses ranged from *not at all reluctant* to *extremely reluctant*), and “Should scholars be discouraged from testing the veracity of this statement?” (responses ranged from *no discouragement* to *very strong discouragement*). The 10 taboo conclusions were as follows:

1. “The tendency to engage in sexually coercive behavior likely evolved because it conferred some evolutionary advantages on men who engaged in such behavior.”
2. “Gender biases are not the most important drivers of the under-representation of women in STEM fields.”
3. “Academia discriminates against Black people (e.g., in hiring, promotion, grants, invitations to participate in colloquia/symposia).”
4. “Biological sex is binary for the vast majority of people.”
5. “The social sciences (in the United States) discriminate against conservatives (e.g., in hiring, promotion, grants, invitations to participate in colloquia/symposia).”
6. “Racial biases are not the most important drivers of higher crime rates among Black Americans relative to White Americans.”
7. “Men and women have different psychological characteristics because of evolution.”
8. “Genetic differences explain non-trivial (10% or more) variance in race differences in intelligence test scores.”
9. “Transgender identity is sometimes the product of social influence.”
10. “Demographic diversity (race, gender) in the workplace often leads to worse performance.”

Participants then reported how at risk they would feel of various consequences (see Table 1) if they shared their views on these topics openly on a sliding scale ranging from *no risk at all* to *very high risk.* (All sliding scales used in our study had a range of 0–100.)

**Table 1.** Questions in Survey

**Possible consequences for sharing views openly**
Being ostracized by some peers
Career-damaging biases against me (e.g., in publishing, promotion, awards, grants, talk invites)
Being stigmatized or labeled pejorative terms
Disciplinary actions (e.g., losing classes, losing leadership roles, formal reprimand)
Guilt-by-association harm to my students and colleagues
Being fired
Being attacked on social media
Student boycotts
Threats of physical violence
**Possible reasons to retract a paper**
Data fraud (i.e., making up or altering data)
Analytic errors that alter primary conclusion
Numerous failures to replicate
Compelling evidence of *p*-hacking (e.g., *p*-curve)
Failure to obtain ethics approval
Moral concerns that the conclusions could harm vulnerable groups
The risk of extremists misconstruing and weaponizing the results
**Possible reasons to fire a scholar**
Data fraud (i.e., making up or altering data)
Numerous failures to replicate their findings
Compelling evidence of *p*-hacking in more than one paper
Engaging in sexual behavior with their own graduate or undergraduate students
Moral concerns about the implications of their research conclusions
Their research has become popular with extremist groups
**Possible petitioner reasons to retract papers**
Data fraud (i.e., making up or altering data)
Research error (e.g., mistake in analysis)
Moral concerns about the conclusions (e.g., findings reinforce negative stereotypes)
**Possible determiners of risk**
University leadership (e.g., presidents, provosts, deans, chairs)
University ethics committees
Journal editors
Peer scholars (e.g., concerns expressed in petitions, social-media campaigns)
The members of the community that the scholar is researching or discussing
Students
The scholar publishing or teaching the research
Nobody—social scientific conclusions should be published and taught regardless of perceived harm risks
**Possible actions against scholars who forward taboo conclusions**
Normal scientific criticism (e.g., commentaries about perceived errors)
Socially ostracizing them
Publicly labeling them pejorative terms (e.g., bigot, racist, sexist)
Disinviting them from talks
Refusing to publish their work regardless of its merits
Not hiring or promoting them even if they meet typical standards
Stigmatizing their graduate students and coauthors
Firing them
Shaming them on social media
Removing them from leadership positions

On a 101-point sliding scale from *definitely not* to *yes,* *definitely*, participants reported whether there are conclusions in the social sciences that have some empirical support but that are nonetheless taboo, such that mentioning supportive evidence would lead to formal or informal punishment. They were also given the option to provide an example in an open-ended response. Participants then evaluated the legitimacy of various reasons to retract articles and fire scholars (see Table 1) on sliding scales ranging from *never legitimate* to *always legitimate*.

Next, participants were asked, “How much admiration vs. contempt do you hold toward peers who start petitions or social media campaigns to retract papers for the following reasons?” and responded on sliding scales ranging from m*aximum contempt* to *maximum admiration* regarding the three reasons provided in Table 1. Participants then reported whether scholars should be completely free to pursue research questions without fear of institutional punishment with response options *no*, *it’s complicated*, and *yes*. And participants reported what scientists should prioritize if pursuit of truth and social-equity goals came into conflict with response options *social equity*, *it’s complicated*, and *truth*. Participants then indicated who should determine whether social scientific conclusions pose too much risk of harm to publish or teach and were able to make multiple selections from the list in Table 1.

Next, participants were asked, “How certain should it be that a social scientific finding is going to cause harm before it should be suppressed?” They were given six response options: *The harm should seem possible*, *The harm should seem likely, There should be suggestive evidence it would cause harm, There should be clear evidence it would cause harm, There should be evidence that the only way to avoid the harm is the suppress the research*, and *We should never suppress social scientific findings.*

Participants were then asked,

In our earlier interviews with psychology professors, we discovered the most taboo conclusions in the social sciences tend to involve genetic or evolutionary explanations for group differences in socially valued outcomes (e.g., education and career outcomes, socioeconomic status, criminal justice involvement), and particularly in domains where women underperform relative to men or where Black people underperform relative to White people. Now imagine a scholar who forwarded a genetic or evolutionary explanation for gender or racial differences in socially valued outcomes in their research. Which of the following actions would you support against him or her?

Participants responded on a sliding scale ranging from *Would not support at all* to *Would strongly support* regarding the 10 actions in Table 1.

Participants were also given an open response box to tell us anything they wanted to share, and last, they reported various demographic details using a sliding scale; options for political ideology ranged from *extremely liberal* to *extremely conservative*. Because of space constraints, some results are reported only in the Supplemental Material.

## Results

We interpreted effects that met a minimum effect-size threshold for a small effect according to Cohen’s standards (|*r*| ≥ .10, |*d*| ≥ 0.2; Cohen, 1977). Because many of our analyses involved comparisons across 10 taboo conclusions, when we refer to statistical significance we use a conservative Bonferroni-corrected *p* value of < .005 (Holland & Copenhaver, 1988). In cases in which we refer to a “small but not significant” effect, we mean that the effect size was |*r*| ≥ .10 or |*d*| ≥ 0.2, but not significant at *p* < .005 (even if it is significant at the more common significance threshold of *p* < .05).

When we report relationships between ideology and other variables, we often report the relationship between conservatism and other variables, with conservatism being on the high end (right side) of the scale. But among the sample, higher conservatism may be characterized as “lower liberalism.” The vast majority of the participants were left of the midpoint, so participants who score higher on conservatism are often more centrist or less liberal than the average participant.

### Taboo beliefs

#### Descriptives

For every conclusion, beliefs ranged from 0 to 100, indicating that some psychology professors were 100% certain the conclusion was true and others 100% certain it was false. As seen in Table 2, average beliefs hovered near the midpoint (±10) for six conclusions, higher (above the midpoint) for (1) evolved psychological sex differences and (2) binary biological sex, and lower (below the midpoint) for (3) genetic contribution to IQ differences and (4) demographic diversity and workplace performance. There was also large variance for most statements, indicating high disagreement, and the majority of professors had at least some uncertainty. These findings seem to indicate little to no scientific consensus on these conclusions, despite high levels of confidence among some scholars (and in both directions).

**Table 2.** Descriptives for Empirical Beliefs, Self-Censorship, and Research Discouragement for Each Taboo Conclusion

Taboo conclusion	Self-censorship			Belief in truth			Discouragement		
	*n*	*M*	*SD*	*n*	*M*	*SD*	*n*	*M*	*SD*
Evolved sexually coercive behavior	467	49.50	32.44	468	53.47	25.77	465	18.24	24.31
Social influence on transgender identity	431	47.02	35.72	429	54.11	29.31	429	11.15	19.98
Racial bias and crime	439	44.09	35.39	438	46.93	29.85	438	7.47	14.82
Binary biological sex	448	42.73	35.13	449	66.10	32.09	447	8.95	16.53
Racial bias in academia	453	41.95	36.99	453	59.29	31.80	452	5.85	12.82
Genetic contribution to IQ differences	434	39.98	39.01	433	29.10	28.94	434	22.54	31.03
Gender bias in STEM	451	38.45	34.72	453	45.26	28.26	450	9.08	16.83
Political bias in social science	445	34.63	31.17	446	52.06	29.55	444	6.22	13.61
Evolved sex differences	439	33.87	31.22	440	65.50	28.49	438	8.71	16.79
Demographic diversity and performance	431	26.33	32.31	432	21.44	23.55	431	8.76	19.31

Note: STEM = science, technology, engineering, and mathematics.

Self-censorship was moderate on average and also ranged from 0 to 100, with many professors reporting no reluctance to share their views and others reporting extreme reluctance. Self-censorship was highest for the evolution of sexually coercive behavior and lowest for the relation between demographic diversity and workplace performance. Scholars generally did not want to discourage research. The most discouragement was observed for a genetic contribution to IQ differences, but the mean was still well below the midpoint. This conclusion also contained the most variance, indicating relatively high disagreement about whether this research should be discouraged.

#### Gender, ideology, and age differences in beliefs

For all gender analyses, we excluded those who did not disclose a gender and the two professors who identified as nonbinary, to protect their responses. As seen in Table 3 and Figure S1 in the Supplemental Material, men believed more strongly in the truth of every single taboo conclusion relative to women, with two exceptions: (a) For political bias in social science, there was a small but not significant effect in the same direction, and (b) women believed more strongly that academia discriminates against Black people. In some cases, differences were quite large. For example, female psychologists (on average) were quite confident that academia discriminates against Black people, but male psychologists (on average) were on the fence; male psychologists (on average) were quite confident that men and women evolved different psychological characteristics, but female psychologists (on average) were on the fence. Future research should explore whether male and female psychology professors present to their students different evidence and arguments regarding the veracity of taboo conclusions.

**Table 3.** Beliefs in Taboo Conclusions by Gender

Taboo Conclusion	*n*	*M*	*SD*	*d*	*t*	*df*	*p*
Evolved sexually coercive behavior				0.37	3.68	402	< .001
Males	238	58.17	24.68				
Females	166	48.94	24.96				
Gender bias in STEM				0.38	3.73	402	< .001
Males	238	49.76	28.85				
Females	166	39.25	26.42				
Racial bias in academia				−0.70	−6.89	401	< .001
Males	237	50.41	32.04				
Females	166	71.29	26.65				
Binary biological sex				0.60	5.91	401	< .001
Males	237	74.60	27.85				
Females	166	56.49	33.42				
Political bias in social science				0.24	2.41	401	.017
Males	237	55.81	30.24				
Females	166	48.75	27.23				
Racial bias and crime				0.30	2.92	399	.004
Males	236	50.28	30.47				
Females	165	41.50	28.43				
Evolved sex differences				0.88	8.70	401	< .001
Males	237	75.36	23.33				
Females	166	52.77	28.70				
Genetic contribution to IQ differences				0.54	5.35	397	< .001
Males	235	35.24	29.87				
Females	164	20.15	24.33				
Social influence on transgender identity				0.37	3.65	398	< .001
Males	236	58.27	27.32				
Females	164	47.58	30.83				
Demographic diversity and performance				0.40	3.95	400	< .001
Males	237	25.00	24.67				
Females	165	15.84	19.96				

Note: For some conclusions variances were not equal, but assuming equal variance (or not) had virtually no impact on the results. STEM = science, technology, engineering, and mathematics.

Female scholars were more left-leaning (*M* = 20.86, *SD* = 16.03) than male scholars (*M* = 27.90, *SD* = 18.70), *t*(401) = 3.93, *p* < .001, and younger, *t*(400) = 4.73, *p* < .001. Conservatism was associated with stronger beliefs that all taboo conclusions are true, *r*s =.19 to .40, *p*s < .001. One exception was a strong association between conservatism and disbelief that academia discriminates against Black people, *r* = −.40, *p* < .001. Age had smaller and more inconsistent associations with belief in the taboo conclusions. When regressing gender, ideology, and age simultaneously on taboo beliefs, with at least small effects, gender continued to predict belief for eight conclusions, ideology continued to predict belief for all ten conclusions, and age predicted belief for three conclusions. These regressions are fully reported in Table S2 in the Supplemental Material.

#### Self-censorship and research discouragement

Conservatism was associated with more self-censorship for all conclusions, *r*s = .20 to .35, *p*s < .001. Conservatism was also associated with less research discouragement with at least small effects for six conclusions, *r*s = −.11 to −.17 (significant at *p* < .005 for only two), but results were trending in the same direction for the other four (gender bias in STEM, racial bias in academia, political bias in social science, evolved sex differences), *r*s = −.06 to −.10, *p*s = .049 to .250.

With at least small effects, in almost every case, males self-censored more than females (*p* < .005 for four conclusions), and females wanted to discourage research more than males (*p* < .005 for seven conclusions; see Table 4 and Figs. S2 and S3 in the Supplemental Material). There were two exceptions: (a) With a small but not significant effect, female professors self-censored more regarding discrimination against conservatives in the social sciences, and (b) male and female professors similarly had almost no desire to discourage research into discrimination against Black people in academia.

**Table 4.** Gender Differences in Self-Censorship and Research Discouragement by Taboo Conclusion

Self-Censorship	*d*	*t*	*df*	*p*
Evolved sexually coercive behavior	0.30	3.01	402	.003
Gender bias in STEM	0.55	5.38	400	< .001
Racial bias in academia	0.39	3.88	401	< .001
Binary biological sex	0.20	1.95	401	.052
Political bias in social science	−0.24	−2.36	401	.019
Racial bias and crime	0.23	2.26	400	.024
Evolved sex differences	0.26	2.57	401	.011
Genetic contribution to IQ differences	0.35	3.44	398	< .001
Social influence on transgender identity	0.24	2.36	399	.019
Demographic diversity and performance	0.26	2.61	400	.010
Desires to discourage research				
Evolved sexually coercive behavior	−0.53	−5.19	400	< .001
Gender bias in STEM	−0.30	−2.94	399	.003
Racial bias in academia	−0.05	−0.51	401	.610
Binary biological sex	−0.29	−2.85	401	.005
Political bias in social science	−0.37	−3.68	401	< .001
Racial bias and crime	−0.37	−3.60	399	< .001
Evolved sex differences	−0.41	−4.01	401	< .001
Genetic contribution to IQ differences	−0.46	−4.56	398	< .001
Social influence on transgender identity	−0.35	−3.40	398	< .001
Demographic diversity and performance	−0.23	−2.24	400	.026

Note: For most outcomes variances were not equal, but assuming equal variance (or not) had virtually no impact on the results. STEM = science, technology, engineering, and mathematics.

When gender, ideology, and age were simultaneously regressed on self-censorship, with at least small effects, gender continued to predict self-censorship for seven conclusions (*p* < .005 only for one), ideology predicted self-censorship for all ten conclusions (*p* < .005 for all), and age predicted self-censorship for four conclusions (*p* < .005 for only two). When gender, ideology, and age were simultaneously regressed on research discouragement, with at least small effects, female gender predicted more discouragement for seven conclusions (*p* < .005 for four), more left-wing ideology predicted more discouragement for only three conclusions (none significant), and younger age predicted more discouragement for seven conclusions (*p* < .005 for five). These regressions are fully reported in Tables S3 and S4 in the Supplemental Material.

**Fig. 1.**
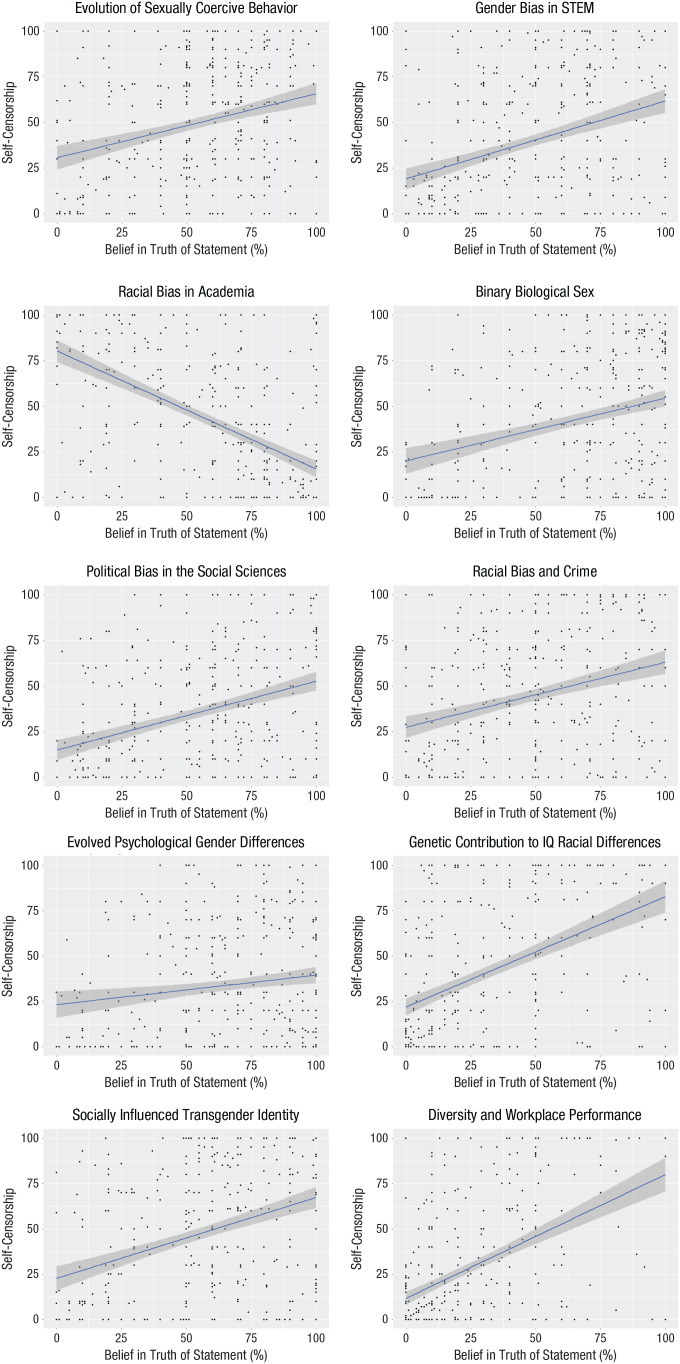
Correlations between taboo beliefs and self-censorship for each taboo conclusion. Higher degrees of self-censorship are along the *y*-axes and stronger belief that the conclusion is true are along the *x*-axes. STEM = science, technology, engineering, and mathematics. The shaded areas represent 95% confidence intervals.

As seen in Figure 1, for nearly all taboo conclusions, scholars who believed the statements were true self-censored more (*r*s = .15–.50, all *p*s < .003). The one exception was for racial bias in academia, for which there was a strong reverse association (*r*s = −.56, *p* < .001). Only two people mentioned this as a taboo conclusion in the pilot study, and in some ways, it opposes the major theme of most other taboo conclusions. To the extent that self-censorship reveals which beliefs are truly taboo, it is perhaps more taboo to suggest that academia does not discriminate against Black people. Consistent with this view, a reliability analysis of all taboo beliefs revealed positive associations between all beliefs, *r*s = .18 to .54, except the belief that academia discriminates against Black people, which was negatively correlated with all other taboo beliefs, *r*s = −.26 to −.45.

Desires to discourage research across topics were positively related, *r*s = .21 to .60, all *p*s < .001. For nearly all taboo conclusions, those who believed the conclusions were false had stronger desires to discourage research, *r*s = −.09 to −.25, *p*s < .048 (*p* < .005 for six). The one exception was again for racial discrimination in academia, but this one was trending in the same direction, *r* = −.08, *p* = .097. These associations indicate that professors with more socially desirable beliefs (i.e., beliefs that taboo conclusions are false) have stronger desires to deter research into taboo topics. Higher self-censorship was associated with more research discouragement for four conclusions, *r*s = .10 to .24, *p*s < .03 (*p* < .005 for three). Table S5 in the Supplemental Material reports all correlations between beliefs, self-censorship, and research discouragement for each conclusion.

### Fear of consequences

When reporting the consequences they would face if they shared their own empirical beliefs openly, professors were quite concerned about getting attacked on social media (*M* = 64.48, *SD* = 33.39), being ostracized by peers (*M* = 54.80, *SD* = 33.22), and being stigmatized or labeled pejorative terms (*M* = 53.34, *SD* = 34.70). Scholars had low to moderate concerns about disciplinary actions (*M* = 32.04, *SD* = 31.96), student boycotts (*M* = 40.69, *SD* = 33.62), guilt-by-association harm to students and colleagues (*M* = 40.89, *SD* = 33.22), and career-damaging biases against them (*M* = 44.79, *SD* = 34.29). These findings suggest that most psychology professors hold some empirical beliefs they consider socially costly.

Scholars were relatively unconcerned about threats of physical violence (*M* = 26.95, *SD* = 28.38) and getting fired (*M* = 17.57, *SD =* 24.69). We computed a “likely tenured” variable by coding associate professors, full professors, and emeritus professors as tenured (*n* = 312) and all other positions as untenured (*n* = 107). Untenured faculty were not significantly more concerned about getting fired (*M* = 20.37, *SD* = 23.91) than tenured faculty (*M* = 16.83, *SD* = 24.93), *t*(417) = 1.28, *p* = .201, *d* = 0.14. This lack of difference could be due to a floor effect, although only 40.4% had no concern about getting fired—and recall that this concern is about whether they would get fired if they shared their own empirical beliefs openly, not a concern about sharing some hypothetical and extreme belief. Thus, the majority of professors hold empirical beliefs that they perceive to be sufficiently socially unwelcome that it would increase risk of termination if others were to discover those beliefs.

There were no differences between the tenured and the untenured on fear of any consequences. We also computed a self-censorship index across all taboo conclusions, ∝ = .92, and found that tenured (*M* = 40.01, *SD* = 26.30) and untenured professors (*M* = 40.55, *SD* = 25.56) self-censor to virtually identical degrees, *t*(410) = 0.16, *p* = .869, possibly because tenure provides no protection against the consequences scholars fear most—ostracism, social-media attacks, and stigmatization.

We computed an index of taboo beliefs. Because belief about discrimination against Black people in academia was negatively related to all other taboo beliefs, we reverse-scored this item, which improved the alpha from .68 to .83. Average taboo beliefs were around the midpoint (*M =* 47.60, *SD* = 18.05, with ~10% in the bottom quartile of the scale and ~7% in the top quartile of the scale). Stronger taboo beliefs, *r*s = .25 to .56, *p*s < .001, and conservatism, *r*s = .19 to .37, *p*s < .001, were associated with higher perceived risks for all consequences.

### Existence of taboos

Scholars generally agreed that some empirically supported conclusions are taboo such that mentioning the supportive evidence would result in punishment (*M* = 66.87, *SD* = 29.56). With a small but not significant effect, men believed that taboo research conclusions exist (*M* = 69.32, *SD* = 30.34) more than women do (*M* = 62.70, *SD* = 28.52), *t*(389) = 2.17, *p* = .031, *d* = 0.22. Conservatism, *r* = .26, *p* < .001, and stronger belief that the taboo conclusions are true, *r* = .50, *p* < .001, were also associated with stronger belief in the existence of taboos.

### Retractions, firings, and attitudes toward retraction petitioners

#### Retractions

Scholars viewed data fraud (*M* = 99.43, *SD* = 5.13) as a highly legitimate reason to retract a paper. Scholars also viewed analytic errors that alter primary conclusions (*M* = 91.12, *SD* = 15.68) and failure to obtain ethics approval (*M* = 80.46, *SD* = 27.37) as highly legitimate. These three criteria are consistent with the Committee on Publication Ethics Council guidelines for retraction (COPE Council, 2019).

Scholars tended to find that compelling evidence of *p*-hacking is a legitimate reason to retract (*M* = 67.47, *SD* = 27.61). By contrast, they tended to report that numerous failures to replicate (*M* = 36.61, *SD* = 30.44), moral concerns that the conclusions could harm vulnerable groups (*M* = 29.61, *SD* = 27.68), and risks of extremists misconstruing and weaponizing results (*M* = 22.11, *SD* = 24.96) are illegitimate reasons to retract.

There were no gender differences in perceived legitimacy of retracting papers on the basis of data fraud, analytic errors, or *p*-hacking, |*d*s| < 0.12, *p*s > .241. With small but not always significant effects, women viewed numerous failures to replicate (*M_women_* = 40.73, *SD* = 30.89; *M_men_* = 34.07, *SD* = 29.99), *t*(402) = −2.17, *p* = .031, *d* = −0.22; failure to obtain ethics approval (*M_women_* = 86.99, *SD* = 21.26; *M_men_* = 76.21, *SD* = 29.83), *t*(402) = −4.00, *p* < .001, *d* = −0.41; moral concerns (*M_women_* = 37.95, *SD* = 29.17; *M_men_* = 24.13, *SD* = 25.19), *t*(402) = −5.08, *p* < .001, *d* = −0.51; and risks that extremists could weaponize the results (*M_women_* = 26.71, *SD* = 25.95; *M_men_* = 19.40, *SD* = 24.05), *t*(402) = −2.91, *p* = .004, *d* = −0.29, as more legitimate reasons than did men. For these four gender differences we reran these as regressions, entering gender, ideology, and age simultaneously, and we report effects that reached minimum thresholds for small effects (although not all were statistically significant). Women (semipartial *r* = .12, *p* = .013) and younger professors (semipartial *r* = .12, *p* = .017) considered numerous failures to replicate a more legitimate reason to retract. For failure to obtain ethics approval only gender was significant, with women endorsing retraction more (semipartial *r* = .16, *p* = .001). Women (semipartial *r* = .19, *p* < .001) and more left-leaning professors (semipartial *r* = −.13, *p* = .006) considered moral concerns a more legitimate reason to retract. And for risks of extremists misconstruing and weaponizing results, only gender predicted retraction support, with women endorsing this reason more (semipartial *r* = .11, *p* = .023).

#### Firings

Scholars considered data fraud (*M* = 95.68, *SD* = 8.54) and sexual behavior with students to be legitimate reasons to fire (*M* = 75.48, *SD* = 26.59). Scholars were on the fence about whether compelling evidence of *p*-hacking in more than one article is a legitimate reason (*M* = 46.78, *SD* = 30.12) to fire a scholar. And scholars generally considered numerous failures to replicate (*M* = 18.67, *SD* = 22.58), moral concerns about research conclusions (*M* = 14.45, *SD* = 19.67), and popularity of research among extremists (*M* = 10.45, *SD* = 15.82) as illegitimate reasons to fire scholars.

Women more highly endorsed firing scholars for all reasons compared to men, |*d*s| ≥ .17, *p*s ≤ .094, but these differences reached minimum thresholds for small effects (and statistical significance at *p* < .005) only for compelling evidence of *p*-hacking (*M_women_* = 53.14, *SD* = 28.43; *M_men_* = 43.23, *SD* = 30.22), *t*(402) = −3.32, *p* < .001, *d* = −0.34; sexual behavior with students (*M_women_* = 81.20, *SD* = 24.03; *M_men_* = 71.10, *SD* = 27.69), *t*(400) = −3.80, *p* < .001, *d* = −0.39; and moral concerns about conclusions (*M_women_* = 17.98, *SD* = 21.87; *M_men_* = 11.90, *SD* = 17.37), *t*(402) = −3.11, *p* = .002, *d* = −0.31.

For these three gender differences we reran these as regressions, entering gender, ideology, and age simultaneously, and we report effects that reached minimum thresholds for small effects (although not all were statistically significant). For compelling evidence of *p*-hacking in more than one article, only gender predicted support for firing, with women endorsing this reason more (semipartial *r* = .15, *p* = .002). Women professors (semipartial *r* = .12, *p* = .014), more left-leaning professors (semipartial *r* = −.14, *p* = .005), and younger professors (semipartial *r* = −.13, *p* = .007) considered sexual behavior with students a more legitimate reason to fire. And women professors (semipartial *r* = .10, *p* = .038) and younger professors (semipartial *r* = −.14, *p* = .006) more highly endorsed firing professors on the basis of moral concerns about their research conclusions.

#### Attitudes toward retractors

**Fig. 2.**
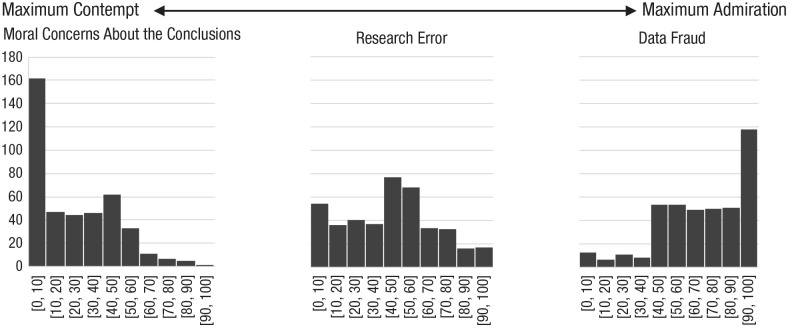
Contempt versus admiration toward peers who start petitions or social-media campaigns to retract papers for moral concerns, research errors, or data fraud. Values on the *y*-axis are frequencies. Bins correspond to values on a response scale ranging from 0 (*maximum contempt*) to 100 (*maximum admiration and respect*).

As seen in Figure 2, scholars generally admired colleagues who participate in social-media campaigns and petitions to retract papers for data fraud (*M* = 71.43, *SD* = 24.78). Professors were more on the fence about peers who participate in retraction campaigns because of research errors, but leaned slightly toward contempt (*M* = 45.50, *SD* = 25.54). And scholars were very contemptuous of peers who participate in retraction campaigns for moral concerns (*M* = 25.91, *SD* = 22.78). This raises the question of whether scholars may be contemptuous of journal editors who retract papers for these reasons.

There were no gender differences in admiration or contempt toward peers who petition to retract papers for data fraud or research errors. However, women had significantly less negative views toward peers who petition to retract papers for moral concerns (*M_women_* = 32.54, *SD* = 23.71; *M_men_* = 21.44, *SD* = 20.61), *t*(398) = −4.98, *p* < .001, *d* = −0.51. When regressing gender, ideology, and age on attitudes toward petitioners to retract for moral concerns, women continued to be less contemptuous (semipartial *r* = .16, *p* < .001), as were more left-leaning and younger professors, (semipartial *r*s = −.19 and −.17, *p*s < .001, respectively).

### Academic freedom and pursuit of truth

A slim majority of professors (52.3%) reported that scholars should be completely free to pursue research questions without fear of institutional punishment for their conclusions. By contrast,1.6% said scholars should not have this freedom, and 46.0% said it’s complicated. Respectively, these values were 60.5%, 2.5%, and 37.0% among men and 39.8%, 0.6%, and 59.6% among women, χ^2^ = 21.03, *p* < .001. Conservatism (*r* = .15, *p* = .002), stronger belief in the veracity of taboo conclusions (*r* = .27, *p* < .001), and self-censorship (*r* = .14, *p* = .004) were associated with higher support for complete academic freedom. When gender, ideology, and age were simultaneously regressed on support for academic freedom, with small but not always significant effects, female professors (semipartial *r* = −.10, *p* = .048), more left-leaning professors (semipartial *r* = .11, *p* = .024), and younger professors (semipartial *r* = .22, *p* < .001) were less supportive of complete academic freedom.

A slim majority of professors (56.5%) reported that scientists should prioritize truth when truth and social-equity goals come into conflict. By contrast, 3.1% prioritized social equity over truth, and 40.5% said it’s complicated. Respectively, these values for men were 66.4%, 1.3%, and 32.4%, and for women, 43.0%, 4.8%, and 52.1% (χ^2^ = 23.37, *p* < .001). Conservatism (*r* = .28, *p* < .001), self-censorship (*r* = .23, *p* < .001), and stronger belief in the veracity of taboo conclusions (*r* = .47, *p* < .001), were associated with increasing prioritization of truth over social equity. When gender, ideology, and age were simultaneously regressed on prioritization of truth, female professors (semipartial *r* = −.15, *p* = .002), more left-leaning professors (semipartial *r* = .23, *p* < .001), and younger professors (semipartial *r* = .18, *p* < .001) were relatively less likely to prioritize truth over social equity.

### Harm threshold for suppression

Regarding the level of harm needed to justify the suppression of research, most scholars reported that either social scientific findings should never be suppressed (41.5%) or that there should be evidence that the only way to avoid the harm is to suppress the research (33.6%). A sizable minority had a lower threshold, believing that there should be clear evidence the research would cause harm (18.0%). Very few scholars wished to suppress research for only suggestive evidence of harm (1.7%), likely harm (4.3%), or possible harm (1.0%). Overall, then, the vast majority of scholars believed that only compelling evidence of harm justifies the suppression of research.

There were little to no gender differences, with men having a very slightly (but not significantly) higher threshold for suppression (*M_men_* = 5.12, *SD* = 1.12; *M_women_* = 4.92, *SD* = 1.01), *t*(396) = 1.77, *p* = .077, *d* = 0.18. More conservative ideology (*r* = .21, *p* < .001), older age (*r* = .14, *p* = .004), and stronger belief in the veracity of taboo conclusions (*r* = .37, *p* < .001) were associated with higher suppression thresholds.

### Actions against taboo scholars

The last question before our open-ended response asked scholars how much they would support various actions against scholars who draw the most taboo conclusions—those that involve genetic or evolutionary explanations for group differences in socially important outcomes (i.e., in domains in which women or Black people underperform relative to men or White people). Scholars very strongly supported normal scientific criticism, such as commentaries (*M* = 92.69, *SD* = 15.96). There was little support for removing such faculty from leadership positions (*M* = 25.37, *SD* = 28.75) and disinviting them from talks (*M* = 22.56, *SD* = 26.91). There was very little support for socially ostracizing them (*M* = 14.62, *SD* = 19.54), publicly labeling them pejorative terms (*M* = 10.18, *SD* = 16.35), refusing to publish their work (*M* = 11.87, *SD* = 19.04), not hiring or promoting them (*M* = 14.65, *SD* = 20.68), stigmatizing their graduate students and coauthors (*M* = 5.34, *SD* = 12.28), firing them (*M* = 6.46, *SD* = 13.68), or shaming them on social media (*M* = 11.01, *SD* = 19.43). Support for normal scientific criticism was either unrelated or negatively related to support for all other punishments (*r*s = −.10–.02), whereas support for all other punishments were positively related (*r*s = .36–.72).

As can be seen in Table 5, there were no gender differences in support for normal scientific criticism or stigmatizing graduate students and coauthors. However, with at least small effects, women were more supportive than men of ostracism, public labeling with pejorative terms, talk disinvitations, refusing to publish work regardless of its merits, not hiring or promoting even if typical standards are met, terminations, social-media shaming, and removal from leadership positions.

**Table 5.** Gender Differences in Support for Actions Against Taboo Scholars

	*d*	*t*	*df*	*p*
Normal scientific criticism (e.g., commentaries about perceived errors)	−0.09	−0.92	398	.356
Socially ostracizing them	−0.34	−3.30	397	.001
Publicly labeling them pejorative terms (e.g., bigot, racist, sexist)	−0.24	−2.34	397	.020
Disinviting them from talks	−0.40	−3.96	396	< .001
Refusing to publish their work regardless of its merits	−0.38	−3.68	396	< .001
Not hiring or promoting them even if they meet typical standards	−0.27	−2.68	395	.008
Stigmatizing their graduate students and coauthors	−0.16	−1.53	396	.128
Firing them	−0.31	−3.05	396	.002
Shaming them on social media	−0.28	−2.78	396	.006
Removing them from leadership positions	−0.50	−4.89	396	< .001

With at least small effects, conservatism was associated with lower support for all actions, *r*s = −.11 to −.28, *p*s < .026, except for stigmatizing graduate students and coauthors, which was trending in the same direction, *r* = −.08, *p* = .094. Greater age was associated with lower support for most punishments, including ostracism, pejorative terms, talk disinvitations, refusal to publish work, failure to hire or promote, dismissal, public shaming, and removal from leadership, *r*s = −.15 to −.22, *p*s *<* .003. Stronger belief in the veracity of the taboo conclusions was associated with lower support for all punishments, *r*s = −.18 to −.45, *p*s < .001, except for normal scientific criticism, *r* = −.01, *p* = .794.

When gender, ideology, and age were simultaneously regressed on support for all actions with at least small effects, females were more supportive of four punishments, more left-leaning professors were more supportive of eight punishments, and younger scholars were more supportive of eight punishments. See Table S6 in the Supplemental Material for full results.

## General Discussion

Several primary findings emerged from this research. There was little scientific consensus about the veracity of numerous controversial research conclusions; positive associations emerged between beliefs in the veracity of taboo conclusions and self-censorship; considerable fear, especially of social sanctions, was reported by psychology professors if they were to share their empirical beliefs openly; and moderate support was expressed for complete academic freedom and the prioritization of truth over social equity. Psychology professors mostly opposed suppressing scholarship on the basis of harm concerns. On average, scholars did not wish to discourage research into any controversial topic; they viewed harm concerns as illegitimate reasons to retract papers or fire scholars, had great contempt for peers who petition to retract papers on moral grounds, and held high evidentiary standards for suppressing science on the basis of harm, with a minimum threshold that there should be clear evidence of harm. As with prior work (Honeycutt et al., 2023; Kaufmann, 2021), we found moderately consistent evidence that women, younger, and more left-leaning scholars tended to be more opposed to controversial research.

Most, but not all, scholars believed that some empirically supported conclusions cannot be mentioned without punishment. Scholars with more socially desirable beliefs were less likely to self-censor, less fearful of punishments, and less likely to perceive the existence of taboos. These patterns may explain why scholars sometimes quarrel about whether academic freedom is at risk—scholars are likelier to notice boundaries when they have crossed them (in their own minds, if not publicly). These patterns of self-censorship also suggest that professional discourse surrounding taboo topics (e.g., at conferences, in faculty meetings, on social media) may be systematically biased toward rejecting taboo conclusions because those who hold taboo empirical beliefs are more likely to remain silent than others.

Most respondents supported complete academic freedom and prioritized truth, but these priorities were associated with more self-censorship, potentially distorting perceptions of the acceptability of controversial research among psychology professors as a group. Most professors were concerned about social ostracization, name-calling, and social-media attacks. This fear might stem from false beliefs about how many scholars endorse such punishments, or it might be a reaction to the small minority that does endorse them. A vocal minority and silent majority may have created a seemingly hostile climate against taboo conclusions and the scholars who forward them, even if the silent majority has great contempt for the vocal minority. Future research should test these possibilities more directly.

Compared to the untenured, tenured professors reported just as much self-censorship and just as much fear of all consequences, including getting fired. Despite relatively low professional risks for tenured professors, reputational costs may still be very high because tenured professors have invested more time and effort into academia and are more entrenched in their professional communities. Reputation might be especially important in academia because success often depends on favorable peer evaluations (e.g., peer review, tenure and promotion decisions, award decisions, talk invitations; Clark, Honeycutt, & Jussim, 2022). Moreover, tenure does not protect against the most feared consequences among psychology professors—ostracism, social-media attacks, and name calling—raising the question of whether tenure promotes academic freedom. Future research should seek to replicate these findings among other samples of professors, including untenured faculty who are not in tenure-track roles. One possibility is that being in a tenure-track role (even while untenured) alleviates some fear of professional consequences compared to people in non-tenure-track roles, but that obtaining tenure within tenure-track roles does not make a large difference.

### Limitations

Perhaps the greatest limitation to the present work is the restricted scope. Professors tend to be busy and protective of their time. To keep the survey short enough to retain participation, we could not ask about all taboo conclusions, nor could we ask all relevant questions about them. Our findings may not generalize to professors at other types of institutions, in other disciplines, or working in other countries. Moreover, our pilot and survey were conducted in early and late 2021, but taboos and the way social groups respond to them change over time and place. For example, recent legislation in some states is posing new threats to academic freedom post-2021. Future research should explore beliefs and attitudes related to taboo scholarship and self-censorship in other disciplines, countries, and years.

Another serious limitation is that the topics of our survey—taboos and self-censorship—regard issues that professors may be reluctant to discuss, even in anonymous online surveys. Consequently, our results may not represent the larger population of psychology professors or even the true attitudes of our sample. With a response rate just over 10%, our findings may underrepresent particular perspectives or particular types of scholars. Among those who did participate, participants may have misrepresented their views or skipped the most controversial questions, which could systematically distort our findings. For example, given that professors who believed taboo conclusions were true reported higher rates of self-censorship, our results might underestimate the degree to which professors believe that the controversial conclusions are true. Our results provide a starting place—preliminary estimates that should be updated as more scholars pursue related questions and collect more data. We hope future researchers will discover better ways of collecting honest reports on potentially controversial viewpoints. Perhaps an old-school approach—distributing unmarked paper surveys at professional conferences and collecting sealed-envelope responses in a dropbox the next day—would more thoroughly alleviate concerns about anonymity and encourage honest responding. This approach also might obtain a higher response rate by allowing researchers to ensure that participation invitations are received by potential participants and not lost in spam folders or overstuffed email inboxes.

### On conflict

This paper identifies numerous sources of disagreement among our peers. It is thus not surprising that many recent policy changes in the behavioral sciences have sparked conflict and controversy (e.g., on social media). We assume that most scholars want what is best for science and broader society, but disagree on what these goals entail and how to achieve them. How can these conflicts be better adjudicated? To resolve empirical disputes and more thoroughly incorporate a diverse range of perspectives, scholars can engage in adversarial collaborations (Clark, Costello, et al., 2022; Clark & Tetlock, 2022). Adversarial collaborations might reduce interpersonal conflict by turning adversaries into sparring partners who improve one another’s science. Adversarial collaborations can also reduce the use of inflammatory modes of dispute resolution—such as straw-manning and ad hominem attacks—that contribute to a needlessly hostile scientific climate and may contribute to professors’ fears of social sanctions.

To reduce normative conflicts, scholars could test the empirical assumptions underlying their moral and value disputes. What are the actual consequences of disseminating potentially harmful scientific conclusions, and what are the consequences of suppressing those conclusions? Recent work suggests that scientific harms are overestimated (Clark, Graso, et al., 2023), but this area needs further exploration. Scholars have proposed numerous possible consequences of suppressing science, such as reduced public trust in science, the emergence of counterproductive interventions and conspiracy theories, reduced social cohesion among scientists and the public, and stifled scientific progress (Clark, Jussim, et al., 2023), but these possible consequences have yet to be explored systematically. Metascientific research on the attitudes, beliefs, and behaviors of scientists themselves—and the associated consequences—would be highly valuable. Such studies are rare, yet scientists’ views are disproportionately consequential for science (and thus for society). Scholars are often busy and reluctant to participate in research, so to incentivize participation, researchers can offer consortia coauthorship (e.g., Schaerer et al., 2023). Alternatively, research participation could be considered a component of service worthy of inclusion on CVs and annual reports.

## Conclusion

Disagreements and debates can be productive in science, particularly when participants are equipped and required to make their case with data. But many scientific conflicts regard normative questions about scientific policy and procedures about which scholars have irreconcilable values. In the present article, we document both empirical and normative disagreements among a sample of U.S. psychology professors. These data cannot resolve the identified conflicts, but they may contribute to a shared understanding of the diversity and distribution of perspectives among psychological scientists.

## Supplemental Material

sj-docx-1-pps-10.1177_17456916241252085 – Supplemental material for Taboos and Self-Censorship Among U.S. Psychology Professors Supplemental material, sj-docx-1-pps-10.1177_17456916241252085 for Taboos and Self-Censorship Among U.S. Psychology Professors by Cory J. Clark, Matias Fjeldmark, Louise Lu, Roy F. Baumeister, Stephen Ceci, Komi Frey, Geoffrey Miller, Wilfred Reilly, Dianne Tice, William von Hippel, Wendy M. Williams, Bo M. Winegard and Philip E. Tetlock in Perspectives on Psychological Science

## Transparency

*Action Editor:* Tim Pleskac

*Editor:* Interim Editorial Panel

*Declaration of Conflicting Interests*

The author(s) declared that there were no conflicts of interest with respect to the authorship or the publication of this article.

**Open Practices:** This study was not preregistered. The Qualtrics survey with verbatim study materials is included in the Supplemental Material. To encourage honest responding, we assured participants that all demographic variables and open-ended responses would be removed from the data file before we shared it publicly; with these variables removed, the remaining portion of our data set (quantitative responses to the primary survey questions) is available on our Open Science Framework (OSF) page.

## Notes

1. The two papers by von Hippel and Buss concerned just one sample, and the one paper by Inbar and Lammers included two samples; hence, there were four comparison samples total.
