# Supplementary material for: Taboos and Self-Censorship Among U.S. Psychology Professors
Source: Perspect Psychol Sci. 2024 May 16;20(5):941–57. doi: 10.1177/17456916241252085 (PMC12408927; doi:10.1177/17456916241252085)
Supplement: sj-docx-1-pps-10.1177_17456916241252085 – Supplemental material for Taboos and Self-Censorship Among U.S. Psychology Professors [file sj-docx-1-pps-10.1177_17456916241252085.docx]

**Supplement for: Taboos and Self-Censorship Among U.S. Psychology Professors**

**(Clark et al., 2024)**

**Table of Contents**

p. 2 Pilot Study

p. 17 List of Included Universities

p. 18 Representativeness Checks

p. 26 Figure S1. Gender Differences in Taboo Beliefs Among Psychology Professors

p. 27 Figure S2. Gender Differences in Self-Censorship by Taboo Conclusion

p. 28 Figure S3. Gender Differences in Research Discouragement by Taboo Conclusion

p. 29 Table S2. Regressing Gender, Ideology, and Age on All Taboo Beliefs

p. 31 Table S3. Regressing Gender, Ideology, and Age on All Self-Censorship

p. 33 Table S4. Regressing Gender, Ideology, and Age on All Research Discouragement

p. 35 Table S5. Correlations Between Belief in Truth, Self-Censorship, and Discouragement for Each Taboo Conclusion

p. 37 Table S6. Regressing Gender, Ideology, and Age on Support for Actions Against Scholars

p. 39 Additional Results

p. 40 Main Study Qualtrics Survey

**Pilot Study**

**Method**

**Participants.** For inclusion, participants had to have a PhD in Psychology or a related discipline and work in the United States. The lead author emailed potential participants asking if she could interview them for a study aimed at understanding the intellectual environment in academia. Participants were selected in three ways. An initial set of intellectually diverse psychology faculty were selected either (1) by the research team to represent competing perspectives related to academic freedom and controversial research or (2) by randomly choosing a letter of the alphabet, visiting a university psychology faculty webpage, and then selecting a faculty member whose last name started with that letter (or the letter closest to it). However, the majority of participants were recruited using a method we call the “adversarial snowball,” in which we asked current participants (at the conclusion of their interview) to recommend other scholars whom they believed would disagree with them on the issues discussed in the interview. This was done to ensure we captured a diverse range of perspectives. The only constraint on the “adversarial snowball” recruitment approach was that nominated scholars had to have a PhD in psychology or a related discipline, which resulted in the inclusion of some scholars outside of psychology and some not currently in faculty positions (details reported below). Over the course of the study, 100 people were emailed and 41 agreed to participate.

Participants (*M*_age_ = 44.37, *SD* = 13.93; 23 male, 17 female, 1 mostly female) generally identified as politically liberal. Mean political ideology on a 100-point scale from 1 = *Extremely liberal* to 100 = *Extremely conservative* was 31.31 (*SD* = 15.96). The most liberal participant responded a 2 and the most conservative responded a 60. On job title, 10 participants identified as assistant professors, 10 as associate professors, 12 as professors, 2 as distinguished professors, 1 as professor emeritus, and 6 as research scientists, research fellows, or postdocs. Most participants were affiliated with psychology departments (n = 33); the remainder were affiliated with departments in related disciplines (business, management, economics, environmental studies, political science, law, sociology). Participants reported race and/or ethnicity in an open response (i.e., participants could self-identify as multiple races or ethnicities at any level of specificity). There were 10 Asian, Asian American, South Asian, Indian, East Asian, or Taiwanese American participants, 5 Black or African American participants, 5 Hispanic participants, 5 Jewish or Ashkenazi Jewish participants, 2 Middle Eastern or Arab participants, 2 Native American or Plains Indian participants, and 22 White, Caucasian, or European participants. This sample almost certainly was not perfectly representative of the population of U.S. psychology faculty, but it was quite diverse, and the mean ideology is similar to the mean ideology in our main study and to other samples from similar populations (e.g., Buss & von Hippel, 2018; Inbar & Lammers, 2012).

**Procedure.** Interviews took place over Zoom. To reassure participants that their identities were protected, responses were documented only with feverish note taking and not video or audio recordings. Although interviews were not timed, it was estimated that interviews ranged from around 15 minutes to around 90 minutes with a mean around one hour. This large variation resulted from a combination of some participants responding in ways that directed them to a shorter version of the interview and some participants having a lot to say.

After participants agreed to participate, they were asked, (1) “Are there certain topics, research questions, or empirical conclusions that you believe are off-limits in your field? In other words, are there certain topics you could study, research questions you could ask, or empirical conclusions you could come to, that if you pursued them or reported data supporting them, you would be formally or informally punished or ostracized by your peers?” and then (2) “And would you say that is generally a good thing or bad thing?”.

If participants said “yes” or some variation on “yes” (e.g., “maybe not entirely off-limits, but some topics are more sensitive than others”) to the first question, they were then asked the follow-up questions in order, (3) “What would you say is the most taboo topic in the field? Or the topic that is subject to the most formal or informal punishment or ostracization if studied?”, (4) “Is the entire topic off limits or is it a particular conclusion within the topic that is off limits? And if conclusion, what conclusion is off limits?”, (5) “If a scholar draws this conclusion (or studies this topic), what penalties should they face, if any?”, (6) (only if they responded “conclusion” to Question 4) “On a 0 to 100 scale, do you believe that that conclusion might be empirically true, 0 being ‘I am 100% certain that it is untrue’ and 100 being ‘I am 100% certain it is true’? With 50 meaning both are equally plausible.”, (7) “On a 0 to 100 scale, how often do you keep your views on that topic to yourself and avoid sharing them openly with your peers and the public, 0 being ‘I never self-censor’ and 100 being ‘I self-censor all the time’?”, (8) “If you were to speak completely openly on this topic, what consequences, if any, would you fear?”, (9) “If scientists could study this topic completely freely, what would be the consequences for science and broader society?”. These participants were then given the option to identify the second most taboo topic and answer the same set of follow-up questions regarding that topic. The first 16 participants were given the option to repeat this process for up to five taboo topics, but this was proving to be quite time intensive, and so after the first 16 participants, participants only repeated this process for up to two taboo topics and then were given the option to list other taboo topics without answering any follow-up questions.

Only three participants said that no topics were taboo or off-limits, and these participants were directed to the question, “Are there certain topics, research questions, or empirical conclusions that you wish were off-limits in your field?”. Follow-up questions were prepared for those who might respond “yes” to this question, but nobody did.

All participants were then directed to a set of more general questions: (10) “In general, do you wish scholars had more or less freedom to study topics without fear of negative personal or professional consequences? Or is it just right?”, (11) “In general, how much do you worry that social science findings can be used to harm others? On a 1 to 7 scale from Not at all to A great deal”, (12) “Which cases from history most clearly demonstrate how accepting or ignoring social science findings can cause concrete harm or benefits? Or are there no cases?”, (13) “On a 0 to 100 scale, when other scholars start a petition or a Twitter campaign to get a paper retracted or get another scholar fired for harmful research, how much do you admire or respect the efforts of those scholars from 0 ‘Maximum contempt’ to 100 ‘Maximum admiration and respect’?”, and last (14) “Is there anything else you would like to tell us?”. Participants also reported a variety of demographics at the end.

**Coding and analyses.** Numeric responses were treated as quantitative data with traditional descriptive and inferential statistics. For all open-ended questions, the interviewer read through all responses to each individual question, identified common themes, and then went back through each response to that question and coded for the presence of those themes. These themes are reported as percentages of participants who mentioned them, and the variety of ways these themes were conveyed are often included in text (i.e., we use participants’ language as much as possible). Because this was only a pilot to gather insights to design the larger survey, not all questions were analyzed.

**Results**

Thirty-two participants (78%) reported that there were topics or conclusions that are off-limits. An additional five (12.2%) said topics were not necessarily off-limits but that there were topics for which you had to be more “careful,” or “sensitive,” or that were “controversial” and held to “different standards” or that were viewed as “sketchy.” One participant (2.4%) said no topics were off-limits; one said none were off limits but that mentors had warned them to be careful about certain topics; and one said not in law but that you find more criticism in social psychology relative to law and economics. These latter three participants were coded as “no” on this question and excluded from the follow-up questions about the most taboo topics and conclusions.

Among those who reported that certain topics or conclusions were off-limits, 72.7% said this was bad,^^[[1]](#footnote-1)^^ 6.1% said it was good, and 21.2% said it was both good and bad, neither good nor bad, a mix, a delicate balance, a two sided-coin, or that it depends. These latter types of responses are henceforth referred to as neither/both. Among those who reported that topics and conclusions were not necessarily off-limits but that some topics were more sensitive, 100% responded neither/both. Among the three participants who reported that there were no off-limits topics, two said it was good, and one said neither/both.

**The most taboo topic.** Among the 92.7% of participants who reported that there were taboos surrounding certain topics or that certain topics were particularly sensitive or controversial, all but one reported that particular conclusions within the topic were off limits rather than the entire topic. Overwhelmingly, the most common theme mentioned for the most taboo topic was group differences, with 86.8% of participants mentioning something about group differences. All of these participants also specified other details about what kinds of group differences are off-limits: 76.3% mentioned race differences; 28.9% mentioned sex or gender differences; 47.4% mentioned differences in IQ, intelligence, cognitive skills, intellectual domains or pursuits, or smartness; 44.1% mentioned differences in other domains such as general abilities and competencies or general achievement, school and academic performance and educational outcomes, work and occupational performance, self-control, criminality and police contact, mating behaviors or attraction, professions, sprinting speed, socioeconomic status, violence, racial biases and hate crime rates, any socially or societally valued outcomes or traits, or any unipolar behavior or outcome where it is easy to assign a good and bad end; 57.9% mentioned that the most taboo conclusions pointed to genetic, biological, evolved, natural, innate, inherent, intrinsic, internal, essentialist, immutable, concrete, immovable, or nativist causes or explanations; 23.7% mentioned that the most taboo conclusions are those that could be perceived as blaming the group or victim, perpetuating isms, stigmatizing, confirming racist stereotypes, justifying future or past harms or different treatment, or attributing fault or responsibility because of a group’s behavior or culture; 21.1% contrasted the off-limits causal explanations to accepted causal explanations including society, social environments or socialization, structuralist frameworks, discrimination, oppression, trauma, bad schools, nurture, or blank slatism; 60.5% mentioned that it is a particular group that cannot be portrayed negatively with 47.4% mentioning Black people or African Americans, 13.2% mentioning women, and 31.6% mentioning broader categories such as oppressed or vulnerable groups or victims of oppression, non-white people or people of color, racial or ethnic minorities, underrepresented minorities, or racial groups with subordinate positions (additionally, one person mentioned same-sex households, one mentioned gay people, and one mentioned Aboriginal Australians).

There were also a handful of more unique responses. Four mentioned LGBTQ, trans issues, gender identity, or anything that suggests transgenderism is short-lived or that transitioning is in any way negative; three mentioned political differences, any findings that clash with the ideology of the department, or positive claims about political conservatives; three mentioned finding that the police are not racist, that society is less racist today than in the past, or anything that opposes anti-racism; two mentioned stereotyping and prejudice, racism, and/or sexism and how academia is sexist and/or racist; one person mentioned anything that challenges diversity, equity, and inclusion initiatives; one mentioned anything that suggests that less aggressive climate mitigation might be beneficial; one mentioned the finding that childhood sexual abuse is not universally experienced as negative; one mentioned the conclusion that people have agency (in contrast to causal forces working top-down from power structures); and one mentioned the evolutionary advantages of abusive behavior.^^[[2]](#footnote-2)^^

***Penalties for drawing the most taboo conclusion.*** Regarding the penalties scholars believed their peers *should* face if they drew the most taboo conclusion, 36.8% said there should be no penalties; 23.7% said there should be no penalties so long as the work was done well or in good faith; 28.9% said the only penalties should be normal scientific criticism (e.g., replies, commentaries, debate); 10.5% said there should be more severe penalties including required diversity training, condemnation or being “called out,” not being hired, non-renewed contracts, demotion, firing, denied funding, denial of certain roles or firing from those roles; 13.2% specified other conditions or consequences, with three suggesting harsher penalties and criticisms should apply to normative (rather than descriptive) claims or to scholars who are public intellectuals or political actors, one reporting indifference to whether interpersonal punishments are appropriate, and one suggesting having real trials with legitimate rules and regulations (rather than self-appointed data police) and that repeated patterns of bad scholarship should matter. One said they did not know.

***Beliefs and self-censorship on the most taboo conclusion.*** Regarding whether participants thought the most taboo conclusion was empirically true, responses ranged from 0% (100% certainty it is false) to 100% (100% certainty it is true), with a mean right at the midpoint (50.9%); 16.2% of participants did not, could not, or would not answer the question; one participant provided no numeric response but answered “not necessarily false”; and one participant specified a large confidence interval around their estimate.^^[[3]](#footnote-3)^^

Regarding how much participants self-censor their own views on the taboo topic or conclusion, responses ranged from 0 (never self-censor) to 100 (always self-censor), with a mean just below the midpoint (42.5); 7.9% said they can’t answer, that they neither express nor self-censor because they have no views on the issue, or that they don’t self-censor but are strategic in how they discuss the issue. The correlation between participants’ views of whether the taboo conclusion was likely empirically true and how much they self-censor was *r* = .60, *p* < .001, indicating that scholars who believed the taboo conclusion might be empirically true were far more likely to self-censor their empirical beliefs than those who believed the taboo conclusion is likely false.

***Feared consequences of speaking freely about the most taboo conclusion.*** Regarding the consequences scholars would fear if they were to openly discuss their empirical beliefs about the taboo conclusion, 50% reported harm to their careers including biases against them (e.g., in publishing, grants, hiring and promotion decisions, awards, invitations, and talks), losing classes, being targeted for retractions, losing collaborators, being unwelcome at conferences, and not having their work taken seriously; 21.1% specifically feared getting fired; 39.5% mentioned reputational damage including being branded as sexist, racist, a bigot, homophobic, transphobic, anti-feminist, a bad ally or advocate for diversity, or otherwise being stigmatized, negatively labeled, deemed a pariah, cancelled, or no longer respected; 26.3% reported interpersonal consequences such as being ostracized, socially outcasted, disliked, having colleagues talk behind their back, giving enemies a sling and arrow, not being invited to parties, or general blowback or backlash; 15.8% reported concerns about social media attacks or criticisms or campaigns meant to cause career damage; 10.5% mentioned fear of student responses or students complaining or boycotting classes; 15.8% feared causing harm to others including guilt/stigmatization by association relationships for their colleagues or students, upsetting or offending people, harming genetics research and dissuading scholars from using behavioral genetics methods, or bringing shame upon their family; 7.9% mentioned other harm such as relationship problems, a fractured livelihood, or being unable to send their kids to college; 18.4% mentioned other kinds of consequences, including harassment, being in the news, being misinterpreted or misquoted, feeling shame, general scientific criticism, getting the guillotine (as a joke), and “anything bad that could happen”; 18.4% reported fearing no consequences if they were to speak openly. Of these seven scholars who feared no consequences, two mentioned that it was because they did not believe the taboo conclusion was true; one mentioned that they could discuss things because they were mixed (racially); one mentioned that they do not fear consequences but felt pressure to go with the flow; and one mentioned that they do not self-censor but other people do because they want to seem like “good people” as a form of performative allyship. One person also mentioned the positive consequences of not self-censorsing: they receive a lot of praise and are called “brave” for their candor.

***Scientific and societal consequences.*** Regarding the consequences for science and society if scholars could study the taboo topic freely, 63.2% mentioned more, better, and faster knowledge and better and more rigorous science; 13.2% mentioned better policies and interventions, more empirically informed paths toward closing gaps and reducing isms, or better ability to counsel; 23.7% mentioned harm to vulnerable groups, including perceived vindication of Black inferiority, reinforcing negative stereotypes, perceived justifications of slavery and racism, the science becoming a magnet for racists, that racists would use the science to demonstrate group inferiority, that the social goal to combat racism trumps the science goal to pursue truth, or that the truth of genes and intelligence would generally be awful and lead to bigotry; 13.2% mentioned social tension or conflict, more argument or confrontation, more enemies and polarization in science, or generally awful social effects; 13.2% mentioned general social or societal benefits or greater public recognition of reality, 10.5% said people would continue to reject the science no matter what it showed; and 23.7% mentioned more unique consequences, with one person mentioning each of the following: headaches for administrators and coddling problems, alleviating shame and guilt among people who had sexual relations with adults as children, bad for science, more coalitions to silence people, that it would drive the knuckleheads out of the academy and make it less embarrassing to be an academic, that it would be very bad for the person who took the position, that there are risks to trusting the self-correction process of science, that it would be better to have conversations around how to talk about the science ethically and responsibly than to shut down the science, that people already do study it freely and that controversy attracts scholars, that the existence of controversy shows that the topic is important and should be studied because people care about it, or that conclusions precede science and that science is used as a tool to support conclusions that exist for political reasons and that fields that are too politicized are delegitimized.

**The second most taboo topic.** Thirty-five participants reported a second most taboo topic and/or conclusion (three of the 38 participants who reported that there were taboos were running short on time and so skipped ahead in the interview); all but one reported that particular conclusions within the topic were off limits rather than the entire topic. Once again, the most common theme was group differences, with 55.6% of participants mentioning something about group differences. All of these participants also specified other details about what kinds of group differences are off-limits: 22.2% mentioned race; 38.9% mentioned sex or gender; 13.9% mentioned intelligence, Wonderlic performance, or math ability; 30.6% mentioned other domains such as ambitiousness, self-control, educational or occupational differences, unemployment, income, representation in science, mentorship ability, differences in political power, military performance, criminogenic propensities, violence, aggression, rape, drug use, psychopathy, sports records, or general performance, general cognitive or psychological or personality or behavioral differences, any valenced characteristics or social valued outcomes, or anything negative or bad; 27.8% mentioned that the most taboo conclusions pointed to genetic, evolved, biological, nativist, inherent, or innate causes or explanations; 11.1% contrasted the off-limits causal explanations to accepted causal explanations including prejudice, environment, racist systems, test bias, external attributions, or constructivist explanations; 27.8% mentioned that it is a particular group that cannot be portrayed negatively with 19.4% mentioning women, 5.6% mentioning Black or African American people, 11.1% mentioning other categories such as Hispanics, LGBTQ+ members, or protected classes or marginalized groups.

Another 22.2% of participants said it was particularly taboo when sex differences intersected with transgender issues and forwarded a variety of taboo conclusions surrounding trans issues, including that biological sex is binary or real or important or meaningful, that differences between biological sexes are large, that transgender identity is not authentic and psychologically healthy and permanent, that transwomen break biological women’s sports records, and that biological sex and gender are linked and not social constructs; 8.3% mentioned conclusions perceived as supporting Republicans or conservatives or portraying Republicans or conservatives more favorably than liberals or Democrats; 5.6% of participants mentioned stereotype accuracy; 5.6% mentioned evolutionary advantages of violence or rape.

An additional 25.0% mentioned more unique responses including that sexual interactions between children and adults is less harmful than commonly assumed, that there are potential negative unintended side-effects of affirmative action policies, that psychedelics have benefits, that racism is a public health crisis and still a problem in science and society and that professional associations are complicit in perpetuating racism, intergroup relations among Israelis and Palestinians or other sensitive or oppressed populations, that gender and racial diversity make things worse, or anything that challenges woke or anti-racist beliefs, or that goes against the claims of activists or says that social justice should not be the top priority of social science.

***Penalties for drawing the second most taboo conclusion.*** Regarding what penalties scholars *should* face for drawing the second most taboo conclusion, 64.3% said there should be no penalties; 14.3% specified that there should be no penalties so long as the work is of sufficient quality or done in good faith; 14.3% said nothing other than normal scientific criticism; 7.1% said there should be more severe consequences including that such research should be held to a higher standard or questioned to a greater degree than other research or that it is complicated and the severity of the penalties should vary according to how stupid the descriptive and normative claims are with possible consequences including robust condemnation, denied tenure or promotion or hiring, or a loss of administrative positions; and 17.9% provided other responses such as that such scholars should be applauded, that there should be no consequences but there would be, that penalizing scholars causes a biased set of researchers with bad motives because those without motives would abandon the area, that scholars who do *not* draw the taboo conclusions when the data point to them should be severely penalized, or that it was difficult to answer.

***Beliefs and self-censorship on the second most taboo conclusion.*** Regarding whether participants thought the second most taboo conclusion was empirically true, responses ranged from 20% certainty it is true to 100% certainty it is true, with a mean well-above the midpoint (74.2%), indicating scholars generally thought the second most taboo conclusion was likely to be true. One participant did not answer and one participant provided no numeric response but said they leaned untrue.^^[[4]](#footnote-4)^^

Regarding how much participants self-censor their own views on the taboo topic or conclusion, responses ranged from 0 (never self-censor) to 100 (always self-censor), with a mean again just below the midpoint (43.8). However, for this second most taboo conclusion, there was virtually no relationship between participants’ views of whether the taboo conclusion was likely empirically true and how much they self-censor, *r* = .04.

Feared consequences for studying the second most taboo topic and the consequences for science and society if scholars could freely study the second most taboo topic were not systematically analyzed because responses were quite similar to those for the most taboo topics.

**General questions.** The majority of participants (80%) wished scholars had more freedom to study topics without fear of personal or professional consequences; 20% felt the current amount of freedom was just right; 0% wished scholars had less freedom than the current amount. Regarding how worried scholars were that social scientific findings could be used to harm others, responses ranged from 1 (not at all) to 7 (a great deal), with a mean just below the midpoint (*M* = 3.60).

Regarding feelings toward scholars who start petitions or Twitter campaigns to get papers retracted or to get other scholars fired for harmful research, responses ranged from 0 (maximum contempt) to 100 (maximum admiration and respect), with a mean well below the midpoint (*M* = 19.08), indicating generally unfavorable attitudes toward scholars who use social pressure to retract science. Many participants offered more thorough explanations for their feelings toward these scholars; common themes included that criticism and commentaries and publishing opposing studies and research are appropriate but that calling for firing or retractions is counterproductive, immature, disgraceful, or authoritarian, or that they cannot respect such scholars as scientists. Many scholars also specified that their feelings vary a lot depending on the specific situation, with many saying their feelings are especially negative when the outrage regards the findings or the results of the study or appears to be a morally or politically motivated objection but less negative when the methods are targeted and justifiably so, and veer more positive in cases of outright data fraud and especially repeated instances of fraud. Five participants offered a second answer for retractions or firings targeted for other reasons (including fraud/data fabrication, ethics violations, or sexual misconduct), and these responses were generally quite positive (range 75-100; *M* = 88.00). Some participants also worried that people use methodological critiques to target papers with findings they dislike and asserted that all papers have methodological flaws and these flaws are only considered retraction-worthy when scholars dislike the findings. One participant said they could not answer and three others gave no numeric response but gave rather ambivalent responses indicating that their feelings depend on the case.

**List of Universities Included**

(Top 100 Universities and Universities with The Top 100 Psychology Departments According to *US News* Rankings in 2021)

| Princeton | UC San Diego | Virginia Tech | U Vermont |
| --- | --- | --- | --- |
| Harvard | UC Davis | American | U Illinois Chicago |
| Columbia | William & Mary | Indiana U Bloomington | U Kentucky |
| MIT | Tulane | Yeshiva U | Rutgers Newark |
| Yale | Boston U | Brigham Young | U Albany |
| Stanford | Brandeis | Gonzaga | U South Florida |
| U Chicago | Case Western | Howard | U Wisconsin Milwaukee |
| Penn | UT Austin | Michigan State | Colorado State |
| Cal Tech | U Wisconsin Madison | NC State | George Mason |
| Johns Hopkins | U Georgia | Stevens Tech | Iowa State |
| Northwestern | U Illinois Urbana-Champaign | Texas Christian U | U Arkansas Fayetteville |
| Duke | Lehigh | U Denver | U Tennessee Knoxville |
| Dartmouth | Northeastern | Binghamton U | Baylor |
| Brown | Pepperdine | Elon | Clark U |
| Vanderbilt | U Miami | Marquette | CUNY Graduate |
| Rice | Ohio State | Stony Brook | CUNY Queens |
| Washington St Louis | Purdue | U Buffalo | Ohio U |
| Cornell | Rensselaer Polytechnic | UC Riverside | Rosalind Franklin |
| Notre Dame | Santa Clara | U Iowa | U Alabama Birmingham |
| UCLA | Villanova | U San Diego | U Houston |
| Emory | Florida State | Auburn | U Louisville |
| Berkeley | Syracuse | Arizona | U Maryland Baltimore |
| Georgetown | U Maryland College Park | UC Merced |  |
| U Michigan | U Pittsburgh | UC Santa Cruz |  |
| U Southern California | U Washington | U Delaware |  |
| Carnegie Mellon | Penn State | U Utah |  |
| U Virginia | Rutgers New Brunswick | U Colorado Boulder |  |
| UNC Chapel Hill | U Connecticut | Arizona State |  |
| Wake Forest | Fordham | U Missouri |  |
| NYU | George Washington | U Oregon |  |
| Tufts | Loyola Marymount | U Kansas |  |
| UC Santa Barbara | Southern Methodist | Virginia Commonwealth |  |
| U Florida | Texas A&M | Temple |  |
| U Rochester | U Mass Amherst | Oregon Health and Science |  |
| Boston College | U Minnesota Twin Cities | U Nebraska Lincoln |  |
| Georgia Tech | Worcester Polytechnic | U Oklahoma |  |
| UC Irvine | Clemson | U Texas Southwestern |  |

**Representativeness Checks**

**Method**

We estimated the representativeness our sample in a few ways. In an approach we refer to as R300, we used a random number generator to select 300 random members of our entire population (all psychology faculty that were invited to participate) and then conducted online searches to code them for gender, academic position, and age. Coding an entire random selection from our population provided unbiased estimates of the gender, academic position, and age composition of our entire population. We then compared our sample to the population to estimate whether our sample was possibly skewed in a particular direction (i.e., overrepresenting a particular group). Given that our primary study was conducted in 2021 and our population coding was conducted in 2023, if our sample was generally representative, we should see that our sample was shifted slightly younger and toward lower tier academic positions compared to our population, but that gender should be similar.

Gender was coded based on names, pictures, and pronouns, typically found on faculty webpages or other sources such as LinkedIn. Academic position was typically available on faculty webpages, CVs linked to faculty pages, or LinkedIn. Age was a bit more challenging. We first prioritized coding for year of Bachelor’s degree because most people earn their Bachelor’s degree at approximately age 22. Through a combination of faculty webpages, CVs, LinkedIn, and Facebook, we were able to identify year of Bachelor’s degree for 236 of the 300 psychology faculty. If we could not find year of Bachelor’s degree, we coded instead for year of PhD, considering this a slightly less reliable indicator of a person’s age due to greater variation in age received. We also coded year of PhD if that information was readily available as we were coding year of Bachelor’s in order to estimate average time between Bachelor’s and PhD degrees. We obtained year of PhD for 231 psychology faculty (from similar sources as above as well as Google Scholar searches for faculty members’ dissertations at their degree granting institution). After these strategies, there remained a small number of cases for which we could not identify year of Bachelor’s or PhD. For these remaining faculty members, we conducted searches of public records (such as voter records) with their full name and city (based on the location of their university affiliation). With this approach, we obtained birth year and age for 18 faculty members. Using these three strategies combined, we were able to estimate age for all 300 psychology faculty.

To compute a “best age estimate”, we first computed birth year by subtracting 22 from Bachelor’s year. Next, we subtracted Bachelor’s year from PhD year to estimate the average length of time that passed between the two degrees. The mean was 7.4 years, and the median and mode were both 7 years, thus for participants with Bachelor’s year missing but PhD year available, we computed birth year by subtracting 29 from PhD year. For any remaining missing data, we used birth year from public records. In cases of overlapping data, the correlations between age computed from Bachelor’s degree and age computed from PhD degree was .963, *p*<.001 (n=174), and (with very small overlapping samples) the correlations between age from public records and age computed from Bachelor’s and PhD degrees were .999, *p* < .001 (n=7) and .988, *p*<.001 (n=12), respectively.

Given the significance of political ideology to our study results, we also wanted to check representativeness along ideology, but this information was not readily publicly available. To estimate potential ideological skew, we compared our sample ideology to reported ideology in two former reports with similar samples. Buss and von Hippel (2018) and von Hippel and Buss (2017) collected political ideology among a sample of members of the *Society for Experimental Social Psychology* (social psychologists 5+ years post-PhD), and Inbar and Lammers (2012) collected social and economic ideology among two samples of members of the *Society for Personality and Social Psychology*. For the Inbar and Lammers (2012) comparison, we included only the faculty members from their samples and compared our social and economic ideology variables to theirs. Our sample reported political ideology on 101-point scales, von Hippel and Buss (2017) used an 11-point scale, and Inbar and Lammers (2012) used a 7-point scale. To compare across them, we re-analyzed the von Hippel and Buss (2017) data (which were publicly available) and the Inbar and Lammers (2012) data (which were shared over email) and checked the percentage of participants who reported that their ideology was to the left (or more liberal) than the midpoint.

We were also aware of one other paper that used a very similar sample to ours—faculty members from the top 100 research universities from the 2016 U.S. News & World Report Rankings (Causadias et al., 2018). This study did not report participant ideology, but it did report gender composition, so we used this as a secondary check on our gender composition.

**Results**

As can be seen in Table S1, for position, our sample was very similar to our population estimates. Consistent with our sample being representative of our population two years earlier (2021 vs. 2023), our sample had slightly more teaching faculty (5.5% vs. 3.0%) and assistant professors (18.4% vs. 14.3%) (more junior positions) and slightly fewer full professors (47.0% vs. 49.3%) and professors emeriti (2.9% vs. 6.7%) (more senior positions). Our population also included one or two cases each of professors who had retired, passed, or left academia. We saw virtually no difference in the percentage of associate professors (24.6% vs. 24.3%), likely because as two years of faculty cohorts were promoted from assistant professor to associate professor, two years of faculty cohorts were promoted from associate professor to full professor.

For age, we corrected our 2023 estimate to a 2021 estimate (the time when members of our population participated in our study) by subtracting 2 years. All age brackets from our sample were within 3 percentage points of our population estimate. However, younger ages were slightly overrepresented, middle ages were almost perfectly represented, and older ages were slightly underrepresented, with the largest deviation being an underrepresentation of professors age 76 and older (2.9% fewer in our sample than the population estimate). It seems plausible to us that this group of professors would be less likely to participate in online surveys because of partial retirement.

Our sample skewed 3.8% more male than our population estimate and 2.4% less male than the Causadias et al (2018) estimate, suggesting our sample is close to representative for males. Our sample skewed 6.9% less female than the population estimate but only 0.3% less female than the Causadias et al (2018) estimate, suggesting the possibility of a small female underrepresentation in our sample or a small female overrepresentation in our population estimate. Our sample included 3.1% nonbinary, other, or undisclosed gender, whereas we did not identify these alternative gender identities in our population coding. Our sample may have slightly overrepresented these alternatives compared to our population only because this information was missing from our population estimate.

For ideology, our left-of-midpoint percentage was nearly identical to the von Hippel and Buss (2017) percentage (88.5% vs. 89.3%). For social ideology, our left-of-midpoint percentage was nearly identical to the Inbar and Lammers (2012) percentages (91.1% vs 91.9% and 92.0%). For economic ideology, our left-of-midpoint percentage skewed further left than the Inbar and Lammers (2012) estimate (77.4% vs. 62.0% and 69.1%).

| Table S1. Representativeness Checks | | |  |  |  |  |  |  |
| --- | --- | --- | --- | --- | --- | --- | --- | --- |
|  | **Sample** | **Population** |  |  |  |  |  |  |
| **Position Check** | **2021** | **R300 2023** |  |  |  |  |  |  |
| **Teaching** | 5.5% | 3.0% |  |  |  |  |  |  |
| **Asst Prof** | 18.4% | 14.3% |  |  |  |  |  |  |
| **Assoc Prof** | 24.6% | 24.3% |  |  |  |  |  |  |
| **Prof** | 47.0% | 49.3% |  |  |  |  |  |  |
| **Emeritus** | 2.9% | 6.7% |  |  |  |  |  |  |
| **Other** | 1.7% | 1.3% |  |  |  |  |  |  |
| **Retired** |  | 0.7% |  |  |  |  |  |  |
| **Deceased** |  | 0.3% |  |  |  |  |  |  |
| **Left Academia** |  | 0.3% |  |  |  |  |  |  |
|  | **Sample** | **Population** |  |  |  |  |  |  |
| **Age Check** | **2021** | **R300 2021C** |  |  |  | |  |  |
| **26-35** | 10.4% | 8.7% |  |  |  |  |  |  |
| **36-45** | 30.4% | 28.0% |  |  |  |  |  |  |
| **46-55** | 27.5% | 28.3% |  |  |  |  |  |  |
| **56-65** | 17.1% | 16.3% |  |  |  |  |  |  |
| **66-75** | 12.3% | 13.3% |  |  |  |  |  |  |
| **76+** | 2.4% | 5.3% |  |  |  |  |  |  |
|  | **Sample** | **Population** |  |  |  |  |  |  |
| **Gender Checks** | **2021** | **R300 2023** | **Caus** |  |  |  |  |  |
| **Male** | 57.1% | 53.3% | 59.5% |  |  |  |  |  |
| **Female** | 39.8% | 46.7% | 40.1% |  |  |  |  |  |
| **Nonbinary** | 0.5% |  |  |  |  |  |  |  |
| **Other/unknown** | 2.6% |  | 0.3% |  |  |  |  |  |
|  | **Sample** |  | **Sample** | **I&L** | **I&L** | **Sample** | **I&L** | **I&L** |
| **Ideology Checks** | **2021** | **vH&B** | **2021 S** | **S1 S** | **S2 S** | **2021 E** | **S1 E** | **S2 E** |
| **% left of midpoint** | 88.5% | 89.3% | 91.1% | 91.9% | 92.0% | 77.4% | 62.0% | 69.1% |
| **Note.** Population R300 estimates refer to our coding of a random 300 members of our population. This was corrected to year 2021 for the age check. Caus refers to Causadias et al., 2018, vH&B refers to von Hippel and Buss (2017), and I&L refers to Inbar and Lammers (2018). S1 refers to Study 1 and S2 refers to Study 2, whereas 'S' refers to social ideology and 'E' refers to economic ideology. | | | | | | | | |

As one final method of testing the representativeness of our sample, we conducted a series of one-sample *t*-tests comparing the demographics of our primary sample to comparison sample demographic means. This required recoding various variables. In our primary and R300 samples, we created 4-point linear position variables (Assistant Professor, Associate Professor, Professor, Professor Emeritus), excluding all positions that could not be treated as linear. We created binary gender variables (0 = male, 1 = female, else = missing) in both samples. In our R300 sample, we recoded continuous age to correspond with categorical age as in the primary sample. And in our primary sample, the von Hippel and Buss sample, and the Inbar and Lammers samples, we recoded continuous ideology variables into binary variables (0 = midpoint or left of it, 1 = right of midpoint).

With this new coding approach, compared to our R300 sample (*M*_position_ = 2.51, *SD*_position_ = .83; *M*_age_ = 4.14, *SD*_age_ = 1.34; *M*_gender_ = .47, *SD*_gender_ = .50), our primary sample (*M*_position_ = 2.37, *SD*_position_ = .83; *M*_age_ = 3.98, *SD*_age_ = 1.27; *M*_gender_ = .41, *SD*_gender_ = .49), was more junior, *d* = -.17, *p* = .001, younger, *d* = -.13, *p* =.011, and more male, *d* = -.12, *p* = .016, although effect sizes never reached the minimum threshold for “small” effects (Cohen, 1977). Compared to von Hippel and Buss (*M*_ideology_ = .02, *SD*_ideology_ = .15), our primary sample (*M*_ideology_ = .07, *SD*_ideology_ = .26) leaned slightly less far-left, *d* = .19, *p*<.001. Our primary sample (*M*_social_ = .05, *SD*_social_ = .22; *M*_econ_ = .17, *SD*_econ_ = .38) did not significantly differ on social or economic ideology compared to Inbar and Lammers Study 1 sample (*M*_social_ = .04, *SD*_social_ = .19; *M*_econ_ = .19, *SD*_econ_ = .40), |*d*s| <.07, *ps* >.206, or social ideology in Inbar and Lammers Study 2 (*M*_social_ = .05, *SD*_social_ = .22), *d* = .01, *p* = .768. However, our sample did lean slightly more left on economic ideology compared to Inbar and Lammers Study 2 sample (*M*_econ_ = .21, *SD*_econ_ = .41), *d* = -.10, *p* = .042. But again, none of these differences reached a minimum threshold for small effects.

The very small effect for position was expected because two years had passed between our primary data collection and our population coding, and so some members of our sample would have been promoted in that period. However, our sample might have slightly underrepresented older scholars (particularly in the 76+ category) and female scholars. The ideology comparisons are a bit more difficult to interpret given their inconsistencies and that our comparison samples were from different populations (faculty members of two professional societies in social psychology) than our sample (psychology professors at the top 133 universities/psychology departments). But it is possible our sample was slightly more moderate in general, slightly further left on economic issues, and representative on social issues.

Across academic position, age, gender, and ideology, our sample appeared very similar to all other population estimates, and observed differences were small. To the extent that we were able to estimate deviations from representativeness, the following groups may have been slightly underrepresented: professors age 76 and above, females (although males were not clearly overrepresented), professors who were further left in general, and professors who were further right on economic issues. Our sample seemed nearly representative along dimensions of academic position and social ideology. Our sample is almost certainly not *perfectly* representative, but it appears to resemble closely the demographics of our population and does not appear particularly unusual. Please see the general discussion in the main text for more thorough discussion of the likely limitations on representativeness.

**References**

Buss, D. M., & von Hippel, W. (2018). Psychological barriers to evolutionary psychology: Ideological bias and coalitional adaptations. *Archives of Scientific Psychology*, *6*(1), 148-158.

Causadias, J. M., Vitriol, J. A., & Atkin, A. L. (2018). Do we overemphasize the role of culture in the behavior of racial/ethnic minorities? Evidence of a cultural (mis) attribution bias in American psychology. *American Psychologist*, *73*(3), 243-255.

Cohen, J. (1977). *Statistical power analysis for the behavioral sciences*. New York: Academic press.

Inbar, Y., & Lammers, J. (2012). Political diversity in social and personality psychology. *Perspectives on Psychological Science*, *7*(5), 496-503.

von Hippel, W., & Buss, D. M. (2017). Do ideologically driven scientific agendas impede understanding and acceptance of evolutionary principles in social psychology? In J.T. Crawford & L. Jussim (Eds.), *The politics of social psychology* (pp. 7-25). New York: Psychology Press.

**Supplemental Figure S1.** *Gender Differences in Taboo Beliefs Among Psychology Professors*

**Supplemental Figure S2.** *Gender Differences in Self-Censorship by Taboo Conclusion*

**Supplemental Figure S3.** *Gender Differences in Research Discouragement by Taboo Conclusion*

**Supplemental Table S2**

| **Table S2.** *Regressing Gender, Ideology, and Age on All Taboo Beliefs* | | | | | | |  |  |
| --- | --- | --- | --- | --- | --- | --- | --- | --- |
|  |  |  |  |  |  | 95%CI | | Semipartial |
|  | *B* | *SE* | *Beta* | *t* | *p* | Low | High | *r* |
| Evolved Sexually Coercive Behavior | | | |  |  |  |  |  |
| (Constant) | 62.26 | 6.51 |  | 9.57 | <.001 | 49.47 | 75.05 |  |
| **Female** | **-8.24** | **2.61** | **-0.16** | **-3.16** | **.002** | **-13.38** | **-3.11** | **-.15** |
| **Conservatism** | **0.20** | **0.07** | **0.14** | **2.85** | **.005** | **0.06** | **0.34** | **.14** |
| Age | -0.33 | 1.00 | -0.02 | -0.33 | .743 | -2.29 | 1.63 | -.02 |
| Gender Bias in STEM | |  |  |  |  |  |  |  |
| (Constant) | 37.03 | 7.10 |  | 5.22 | <.001 | 23.07 | 50.99 |  |
| **Female** | **-6.24** | **2.85** | **-0.11** | **-2.19** | **.029** | **-11.84** | **-0.63** | **-.10** |
| **Conservatism** | **0.41** | **0.08** | **0.26** | **5.43** | **<.001** | **0.26** | **0.56** | **.26** |
| Age | 1.72 | 1.09 | 0.08 | 1.58 | .114 | -0.42 | 3.86 | .08 |
| Racial Bias in Academia | |  |  |  |  |  |  |  |
| (Constant) | 75.34 | 7.28 |  | 10.36 | <.001 | 61.03 | 89.64 |  |
| **Gender** | **13.40** | **2.91** | **0.21** | **4.60** | **<.001** | **7.67** | **19.13** | **.20** |
| **Conservatism** | **-0.58** | **0.08** | **-0.33** | **-7.43** | **<.001** | **-0.73** | **-0.43** | **-.32** |
| **Age group** | **-5.16** | **1.11** | **-0.21** | **-4.64** | **<.001** | **-7.34** | **-2.97** | **-.20** |
| Binary Biological Sex | |  |  |  |  |  |  |  |
| (Constant) | 68.39 | 7.64 |  | 8.95 | <.001 | 53.37 | 83.41 |  |
| **Female** | **-13.92** | **3.06** | **-0.22** | **-4.55** | **<.001** | **-19.94** | **-7.90** | **-.21** |
| **Conservatism** | **0.52** | **0.08** | **0.30** | **6.36** | **<.001** | **0.36** | **0.68** | **.29** |
| Age | 1.36 | 1.17 | 0.06 | 1.16 | .246 | -0.94 | 3.65 | .05 |
| Political Bias in Social Science | | |  |  |  |  |  |  |
| (Constant) | 42.34 | 7.15 |  | 5.93 | <.001 | 28.29 | 56.39 |  |
| Female | -2.67 | 2.86 | -0.05 | -0.93 | .352 | -8.29 | 2.96 | -.04 |
| **Conservatism** | **0.63** | **0.08** | **0.39** | **8.19** | **<.001** | **0.48** | **0.78** | **.38** |
| Age | -0.34 | 1.09 | -0.02 | -0.31 | .757 | -2.48 | 1.81 | -.01 |
| Racial Bias and Crime | |  |  |  |  |  |  |  |
| (Constant) | 27.91 | 7.58 |  | 3.68 | <.001 | 13.01 | 42.81 |  |
| Female | -3.56 | 3.04 | -0.06 | -1.17 | .242 | -9.53 | 2.41 | -.06 |
| **Conservatism** | **0.42** | **0.08** | **0.25** | **5.11** | **<.001** | **0.26** | **0.58** | **.24** |
| **Age** | **3.35** | **1.16** | **0.14** | **2.90** | **.004** | **1.08** | **5.62** | **.14** |
| Evolved Sex Differences | |  |  |  |  |  |  |  |
| (Constant) | 76.28 | 6.60 |  | 11.56 | <.001 | 63.30 | 89.26 |  |
| **Female** | **-18.95** | **2.64** | **-0.33** | **-7.17** | **<.001** | **-24.15** | **-13.76** | **-.32** |
| **Conservatism** | **0.35** | **0.07** | **0.23** | **5.01** | **<.001** | **0.22** | **0.49** | **.22** |
| Age | 1.89 | 1.01 | 0.09 | 1.88 | .061 | -0.09 | 3.87 | .08 |
| Genetic Contribution to IQ Differences | | | |  |  |  |  |  |
| (Constant) | 14.83 | 7.02 |  | 2.11 | .035 | 1.04 | 28.63 |  |
| **Female** | **-9.56** | **2.81** | **-0.16** | **-3.40** | **<.001** | **-15.08** | **-4.04** | **-.16** |
| **Conservatism** | **0.41** | **0.08** | **0.25** | **5.42** | **<.001** | **0.26** | **0.56** | **.25** |
| **Age** | **4.40** | **1.07** | **0.20** | **4.11** | **<.001** | **2.30** | **6.51** | **.19** |
| Social Influence on Transgender Identity | | | |  |  |  |  |  |
| (Constant) | 50.81 | 7.18 |  | 7.08 | <.001 | 36.70 | 64.93 |  |
| **Female** | **-7.11** | **2.88** | **-0.12** | **-2.47** | **.014** | **-12.77** | **-1.45** | **-.11** |
| **Conservatism** | **0.58** | **0.08** | **0.36** | **7.55** | **<.001** | **0.43** | **0.73** | **.35** |
| Age | -0.38 | 1.10 | -0.02 | -0.35 | .726 | -2.54 | 1.77 | -.02 |
| Demographic Diversity and Performance | | | |  |  |  |  |  |
| (Constant) | 21.46 | 5.71 |  | 3.76 | <.001 | 10.23 | 32.69 |  |
| **Female** | **-6.27** | **2.29** | **-0.13** | **-2.74** | **.006** | **-10.77** | **-1.77** | **-.13** |
| **Conservatism** | **0.45** | **0.06** | **0.35** | **7.36** | **<.001** | **0.33** | **0.57** | **.34** |
| Age | -0.67 | 0.87 | -0.04 | -0.77 | .442 | -2.39 | 1.04 | -.04 |
| Note. Bold indicates \|semipartial r\| ≥ .10. | | | |  |  |  |  |  |

**Supplemental Table S3**

| **Table S3.** *Regressing Gender, Ideology, and Age on All Self-Censorship* | | | | | | | |  |
| --- | --- | --- | --- | --- | --- | --- | --- | --- |
|  |  |  |  |  |  | 95%CI | | Semipartial |
|  | *B* | *SE* | *Beta* | *t* | *p* | Low | High | *r* |
| Evolved Sexually Coercive Behavior | | | |  |  |  |  |  |
| (Constant) | 57.88 | 8.35 |  | 6.94 | <.001 | 41.47 | 74.28 |  |
| **Female** | **-8.38** | **3.35** | **-0.13** | **-2.50** | **.013** | **-14.97** | **-1.79** | **-.12** |
| **Conservatism** | **0.32** | **0.09** | **0.18** | **3.56** | **<.001** | **0.14** | **0.50** | **.17** |
| Age | -1.15 | 1.28 | -0.05 | -0.90 | .371 | -3.66 | 1.37 | -.04 |
| Gender Bias in STEM | |  |  |  |  |  |  |  |
| (Constant) | 55.01 | 8.55 |  | 6.43 | <.001 | 38.20 | 71.82 |  |
| **Female** | **-15.70** | **3.43** | **-0.23** | **-4.58** | **<.001** | **-22.43** | **-8.96** | **-.22** |
| **Conservatism** | **0.47** | **0.09** | **0.25** | **5.16** | **<.001** | **0.29** | **0.65** | **.24** |
| Age | -1.71 | 1.31 | -0.06 | -1.31 | .192 | -4.27 | 0.86 | -.06 |
| Racial Bias in Academia | |  |  |  |  |  |  |  |
| (Constant) | 34.02 | 9.17 |  | 3.71 | <.001 | 15.99 | 52.05 |  |
| **Gender** | **-9.12** | **3.67** | **-0.12** | **-2.48** | **.013** | **-16.35** | **-1.90** | **-.12** |
| **Conservatism** | **0.63** | **0.10** | **0.31** | **6.42** | **<.001** | **0.44** | **0.82** | **.30** |
| Age group | 1.44 | 1.40 | 0.05 | 1.03 | .305 | -1.31 | 4.19 | .05 |
| Binary Biological Sex | |  |  |  |  |  |  |  |
| (Constant) | 52.28 | 8.89 |  | 5.88 | <.001 | 34.81 | 69.75 |  |
| Female | -5.89 | 3.56 | -0.08 | -1.65 | .099 | -12.88 | 1.11 | -.08 |
| **Conservatism** | **0.45** | **0.10** | **0.23** | **4.70** | **<.001** | **0.26** | **0.64** | **.23** |
| **Age** | **-3.29** | **1.36** | **-0.12** | **-2.43** | **.016** | **-5.96** | **-0.63** | **-.12** |
| Political Bias in Social Science | | |  |  |  |  |  |  |
| (Constant) | 23.31 | 7.74 |  | 3.01 | .003 | 8.10 | 38.52 |  |
| **Female** | **8.59** | **3.10** | **0.14** | **2.77** | **.006** | **2.50** | **14.68** | **.13** |
| **Conservatism** | **0.52** | **0.08** | **0.30** | **6.24** | **<.001** | **0.35** | **0.68** | **.30** |
| **Age** | **-3.45** | **1.18** | **-0.14** | **-2.92** | **.004** | **-5.77** | **-1.13** | **-.14** |
| Racial Bias and Crime | |  |  |  |  |  |  |  |
| (Constant) | 43.70 | 8.90 |  | 4.91 | <.001 | 26.20 | 61.20 |  |
| Female | -5.29 | 3.57 | -0.07 | -1.48 | .139 | -12.30 | 1.73 | -.07 |
| **Conservatism** | **0.53** | **0.10** | **0.27** | **5.56** | **<.001** | **0.34** | **0.72** | **.27** |
| Age | -1.65 | 1.36 | -0.06 | -1.21 | .227 | -4.32 | 1.03 | -.06 |
| Evolved Sex Differences | | |  |  |  |  |  |  |
| (Constant) | 55.21 | 7.92 |  | 6.97 | <.001 | 39.64 | 70.77 |  |
| **Female** | **-8.34** | **3.17** | **-0.13** | **-2.63** | **.009** | **-14.57** | **-2.10** | **-.13** |
| **Conservatism** | **0.30** | **0.09** | **0.17** | **3.54** | **<.001** | **0.13** | **0.47** | **.17** |
| **Age** | **-4.37** | **1.21** | **-0.18** | **-3.61** | **<.001** | **-6.74** | **-1.99** | **-.18** |
| Genetic Contribution to IQ Differences | | | |  |  |  |  |  |
| (Constant) | 38.71 | 9.83 |  | 3.94 | <.001 | 19.38 | 58.04 |  |
| **Female** | **-9.76** | **3.94** | **-0.13** | **-2.48** | **.014** | **-17.49** | **-2.02** | **-.12** |
| **Conservatism** | **0.54** | **0.11** | **0.25** | **5.17** | **<.001** | **0.34** | **0.75** | **.25** |
| Age | 0.26 | 1.50 | 0.01 | 0.17 | .865 | -2.70 | 3.21 | .01 |
| Social Influence on Transgender Identity | | | |  |  |  |  |  |
| (Constant) | 60.69 | 9.06 |  | 6.70 | <.001 | 42.88 | 78.51 |  |
| **Female** | **-7.66** | **3.64** | **-0.11** | **-2.11** | **.036** | **-14.81** | **-0.51** | **-.10** |
| **Conservatism** | **0.47** | **0.10** | **0.24** | **4.83** | **<.001** | **0.28** | **0.66** | **.23** |
| **Age** | **-3.68** | **1.38** | **-0.13** | **-2.66** | **.008** | **-6.40** | **-0.96** | **-.13** |
| Demographic Diversity and Performance | | | |  |  |  |  |  |
| (Constant) | 24.14 | 8.15 |  | 2.96 | .003 | 8.12 | 40.15 |  |
| Female | -5.03 | 3.27 | -0.08 | -1.54 | .124 | -11.45 | 1.38 | -.07 |
| **Conservatism** | **0.53** | **0.09** | **0.30** | **6.11** | **<.001** | **0.36** | **0.71** | **.29** |
| Age | -1.00 | 1.24 | -0.04 | -0.81 | .420 | -3.45 | 1.44 | -.04 |
| Note. Bold indicates \|semipartial r\| ≥ .10. | | | |  |  |  |  |  |

**Supplemental Table S4**

| **Table S4.** *Regressing Gender, Ideology, and Age on All Research Discouragement* | | | | | | | | |
| --- | --- | --- | --- | --- | --- | --- | --- | --- |
|  |  |  |  |  |  | 95%CI | | Semipartial |
|  | *B* | *SE* | *Beta* | *t* | *p* | Low | High | *r* |
| Evolved Sexually Coercive Behavior | | | |  |  |  |  |  |
| (Constant) | 20.15 | 6.07 |  | 3.32 | <.001 | 8.22 | 32.08 |  |
| **Female** | **9.54** | **2.44** | **0.20** | **3.92** | **<.001** | **4.75** | **14.33** | **.19** |
| **Conservatism** | **-0.14** | **0.07** | **-0.11** | **-2.18** | **.030** | **-0.27** | **-0.01** | **-.10** |
| **Age** | **-3.09** | **0.93** | **-0.16** | **-3.32** | **<.001** | **-4.91** | **-1.26** | **-.16** |
| Gender Bias in STEM | |  |  |  |  |  |  |  |
| (Constant) | 6.98 | 4.35 |  | 1.60 | .110 | -1.58 | 15.54 |  |
| **Female** | **4.15** | **1.74** | **0.12** | **2.38** | **.018** | **0.72** | **7.57** | **.12** |
| Conservatism | -0.06 | 0.05 | -0.06 | -1.18 | .240 | -0.15 | 0.04 | -.06 |
| Age | -0.72 | 0.67 | -0.06 | -1.09 | .278 | -2.03 | 0.59 | -.05 |
| Racial Bias in Academia | | |  |  |  |  |  |  |
| (Constant) | 7.31 | 3.38 |  | 2.16 | .031 | 0.66 | 13.96 |  |
| Gender | 0.37 | 1.35 | 0.01 | 0.27 | .785 | -2.29 | 3.03 | .01 |
| Conservatism | -0.04 | 0.04 | -0.05 | -1.04 | .299 | -0.11 | 0.03 | -.05 |
| Age group | -0.31 | 0.52 | -0.03 | -0.60 | .548 | -1.33 | 0.70 | -.03 |
| Binary Biological Sex | |  |  |  |  |  |  |  |
| (Constant) | 14.30 | 4.18 |  | 3.42 | <.001 | 6.09 | 22.52 |  |
| Female | 2.91 | 1.67 | 0.09 | 1.74 | .083 | -0.38 | 6.20 | .09 |
| Conservatism | -0.08 | 0.05 | -0.09 | -1.81 | .072 | -0.17 | 0.01 | -.09 |
| **Age** | **-1.97** | **0.64** | **-0.16** | **-3.08** | **.002** | **-3.22** | **-0.71** | **-.15** |
| Political Bias in Social Science | | |  |  |  |  |  |  |
| (Constant) | 5.35 | 3.55 |  | 1.51 | .132 | -1.63 | 12.32 |  |
| **Female** | **4.16** | **1.42** | **0.15** | **2.93** | **.004** | **1.36** | **6.95** | **.14** |
| Conservatism | -0.05 | 0.04 | -0.07 | -1.40 | .161 | -0.13 | 0.02 | -.07 |
| Age | -0.95 | 0.54 | -0.09 | -1.76 | .080 | -2.02 | 0.11 | -.09 |
| Racial Bias and Crime | |  |  |  |  |  |  |  |
| (Constant) | 8.10 | 3.86 |  | 2.10 | .036 | 0.51 | 15.68 |  |
| **Female** | **4.38** | **1.54** | **0.15** | **2.84** | **.005** | **1.35** | **7.42** | **.14** |
| Conservatism | -0.06 | 0.04 | -0.07 | -1.49 | .138 | -0.14 | 0.02 | -.07 |
| **Age** | **-1.34** | **0.59** | **-0.11** | **-2.27** | **.024** | **-2.49** | **-0.18** | **-.11** |
| Evolved Sex Differences | | |  |  |  |  |  |  |
| (Constant) | 9.59 | 4.13 |  | 2.32 | .021 | 1.47 | 17.72 |  |
| **Female** | **5.00** | **1.66** | **0.15** | **3.02** | **.003** | **1.74** | **8.25** | **.15** |
| Conservatism | -0.05 | 0.04 | -0.05 | -1.05 | .294 | -0.13 | 0.04 | -.05 |
| **Age** | **-1.80** | **0.63** | **-0.14** | **-2.86** | **.004** | **-3.05** | **-0.56** | **-.14** |
| Genetic Contribution to IQ Differences | | | |  |  |  |  |  |
| (Constant) | 24.47 | 7.76 |  | 3.15 | .002 | 9.21 | 39.73 |  |
| **Female** | **10.63** | **3.11** | **0.17** | **3.42** | **<.001** | **4.52** | **16.74** | **.17** |
| Conservatism | -0.15 | 0.08 | -0.09 | -1.80 | .073 | -0.31 | 0.01 | -.09 |
| **Age** | **-3.51** | **1.19** | **-0.15** | **-2.96** | **.003** | **-5.84** | **-1.17** | **-.14** |
| Social Influence on Transgender Identity | | | |  |  |  |  |  |
| (Constant) | 14.50 | 5.02 |  | 2.89 | .004 | 4.63 | 24.38 |  |
| **Female** | **4.72** | **2.01** | **0.12** | **2.35** | **.019** | **0.77** | **8.66** | **.12** |
| **Conservatism** | **-0.15** | **0.05** | **-0.14** | **-2.79** | **.005** | **-0.26** | **-0.04** | **-.14** |
| **Age** | **-1.66** | **0.77** | **-0.11** | **-2.17** | **.031** | **-3.17** | **-0.15** | **-.11** |
| Demographic Diversity and Performance | | | |  |  |  |  |  |
| (Constant) | 16.79 | 5.00 |  | 3.36 | <.001 | 6.96 | 26.63 |  |
| Female | 2.47 | 2.00 | 0.06 | 1.23 | .218 | -1.47 | 6.42 | .06 |
| **Conservatism** | **-0.12** | **0.05** | **-0.11** | **-2.16** | **.031** | **-0.22** | **-0.01** | **-.11** |
| **Age** | **-2.19** | **0.76** | **-0.15** | **-2.87** | **.004** | **-3.69** | **-0.69** | **-.14** |
| Note. Bold indicates \|semipartial r\| ≥ .10. | | | |  |  |  |  |  |

**Supplemental Table S5**

| **Supplemental Table S5.** *Correlations between belief in truth, self-censorship, and discouragement for each Taboo Conclusion* | | | |
| --- | --- | --- | --- |
| **Taboo Conclusion** |  | **Belief in truth** | **Self-censorship** |
| Evolved Sexually Coercive Behavior |  |  |  |
| **Self-censorship** | *r* | **0.28** |  |
|  | *p* | <.001 |  |
|  | *n* | 467 |  |
| **Discouragement** | *r* | **-0.23** | 0.07 |
|  | *p* | <.001 | .158 |
|  | *n* | 465 | 464 |
| Gender Bias in STEM |  |  |  |
| **Self-censorship** | *r* | **0.35** |  |
|  | *p* | <.001 |  |
|  | *n* | 451 |  |
| **Discouragement** | *r* | **-0.14** | 0.05 |
|  | *p* | .002 | .272 |
|  | *n* | 450 | 449 |
| Racial Bias in Academia |  |  |  |
| **Self-censorship** | *r* | **-0.56** |  |
|  | *p* | <.001 |  |
|  | *n* | 453 |  |
| **Discouragement** | *r* | -0.08 | **0.15** |
|  | *p* | .097 | .001 |
|  | *n* | 452 | 452 |
| Binary Biological Sex |  |  |  |
| **Self-censorship** | *r* | **0.31** |  |
|  | *p* | <.001 |  |
|  | *n* | 448 |  |
| **Discouragement** | *r* | **-0.15** | **0.10** |
|  | *p* | .002 | .029 |
|  | *n* | 447 | 447 |
| Political Bias in Social Science |  |  |  |
| **Self-censorship** | *r* | **0.36** |  |
|  | *p* | <.001 |  |
|  | *n* | 445 |  |
| **Discouragement** | *r* | **-0.09** | **0.14** |
|  | *p* | .047 | .003 |
|  | *n* | 444 | 444 |
| Racial Bias and Crime |  |  |  |
| **Self-censorship** | *r* | **0.30** |  |
|  | *p* | <.001 |  |
|  | *n* | 438 |  |
| **Discouragement** | *r* | **-0.10** | 0.03 |
|  | *p* | .036 | .550 |
|  | *n* | 437 | 438 |
| Evolved Sex Differences |  |  |  |
| **Self-censorship** | *r* | **0.15** |  |
|  | *p* | .002 |  |
|  | *n* | 439 |  |
| **Discouragement** | *r* | **-0.23** | **0.24** |
|  | *p* | <.001 | <.001 |
|  | *n* | 438 | 438 |
| Genetic Contribution to IQ Differences | |  |  |
| **Self-censorship** | *r* | **0.45** |  |
|  | *p* | <.001 |  |
|  | *n* | 432 |  |
| **Discouragement** | *r* | **-0.24** | -0.03 |
|  | *p* | <.001 | .563 |
|  | *n* | 432 | 433 |
| Social Influence on Transgender Identity | |  |  |
| **Self-censorship** | *r* | **0.37** |  |
|  | *p* | <.001 |  |
|  | *n* | 429 |  |
| **Discouragement** | *r* | **-0.25** | 0.06 |
|  | *p* | <.001 | .208 |
|  | *n* | 428 | 429 |
| Demographic Diversity and Performance | |  |  |
| **Self-censorship** | *r* | **0.50** |  |
|  | *p* | <.001 |  |
|  | *n* | 431 |  |
| **Discouragement** | *r* | **-0.11** | 0.04 |
|  | *p* | .019 | .400 |
|  | *n* | 431 | 431 |

**Supplemental Table S6**

| **Table S6.** *Regressing Gender, Ideology, and Age on Support for Actions Against Scholars* | | | | | | | | |
| --- | --- | --- | --- | --- | --- | --- | --- | --- |
|  |  |  |  |  |  | 95%CI | | Semipartial |
|  | *B* | *SE* | *Beta* | *t* | *p* | Low | High | *r* |
| Normal scientific criticism (e.g., commentaries about perceived errors) | | | | | | | |  |
| (Constant) | 95.88 | 4.23 |  | 22.65 | <.001 | 87.56 | 104.20 |  |
| Female | 0.61 | 1.70 | 0.02 | 0.36 | .720 | -2.73 | 3.95 | .02 |
| **Conservatism** | **-0.09** | **0.05** | **-0.10** | **-1.96** | **.051** | **-0.18** | **0.00** | **-.10** |
| Age | -0.43 | 0.65 | -0.04 | -0.67 | .503 | -1.71 | 0.84 | -.03 |
| Socially ostracizing them | | |  |  |  |  |  |  |
| (Constant) | 22.01 | 4.95 |  | 4.45 | <.001 | 12.27 | 31.74 |  |
| Female | 3.84 | 1.99 | 0.10 | 1.93 | .054 | -0.07 | 7.74 | .09 |
| **Conservatism** | **-0.18** | **0.05** | **-0.17** | **-3.41** | **<.001** | **-0.29** | **-0.08** | **-.17** |
| **Age** | **-2.10** | **0.76** | **-0.14** | **-2.78** | **.006** | **-3.59** | **-0.61** | **-.14** |
| Publicly labeling them pejorative terms (e.g., bigot, racist, sexist) | | | | | | |  |  |
| (Constant) | 17.16 | 4.24 |  | 4.05 | <.001 | 8.82 | 25.50 |  |
| Gender | 2.01 | 1.70 | 0.06 | 1.18 | .239 | -1.34 | 5.35 | .06 |
| **Conservatism** | **-0.11** | **0.05** | **-0.12** | **-2.43** | **.015** | **-0.20** | **-0.02** | **-.12** |
| **Age group** | **-1.79** | **0.65** | **-0.14** | **-2.76** | **.006** | **-3.06** | **-0.51** | **-.14** |
| Disinviting them from talks | | |  |  |  |  |  |  |
| (Constant) | 39.23 | 6.67 |  | 5.88 | <.001 | 26.12 | 52.33 |  |
| **Female** | **5.64** | **2.68** | **0.10** | **2.11** | **.036** | **0.38** | **10.91** | **.10** |
| **Conservatism** | **-0.36** | **0.07** | **-0.25** | **-5.11** | **<.001** | **-0.50** | **-0.22** | **-.24** |
| **Age** | **-3.88** | **1.02** | **-0.19** | **-3.81** | **<.001** | **-5.88** | **-1.88** | **-.18** |
| Refusing to publish their work regardless of its merits | | | | | |  |  |  |
| (Constant) | 14.53 | 4.96 |  | 2.93 | .004 | 4.78 | 24.27 |  |
| **Female** | **5.17** | **1.99** | **0.13** | **2.60** | **.010** | **1.26** | **9.09** | **.13** |
| **Conservatism** | **-0.12** | **0.05** | **-0.12** | **-2.34** | **.020** | **-0.23** | **-0.02** | **-.12** |
| **Age** | **-1.71** | **0.76** | **-0.11** | **-2.26** | **.025** | **-3.20** | **-0.22** | **-.11** |
| Not hiring or promoting them even if they meet typical standards | | | | | | |  |  |
| (Constant) | 22.88 | 5.42 |  | 4.22 | <.001 | 12.22 | 33.54 |  |
| Female | 3.23 | 2.18 | 0.08 | 1.48 | .139 | -1.06 | 7.51 | .07 |
| **Conservatism** | **-0.15** | **0.06** | **-0.13** | **-2.51** | **.013** | **-0.26** | **-0.03** | **-.12** |
| **Age** | **-2.28** | **0.83** | **-0.14** | **-2.75** | **.006** | **-3.90** | **-0.65** | **-.14** |
| Stigmatizing their graduate students and co-authors | | | | | |  |  |  |
| (Constant) | 5.14 | 3.29 |  | 1.56 | .120 | -1.34 | 11.61 |  |
| Female | 1.48 | 1.32 | 0.06 | 1.12 | .266 | -1.13 | 4.08 | .06 |
| Conservatism | -0.05 | 0.04 | -0.07 | -1.27 | .205 | -0.11 | 0.03 | -.06 |
| Age | -0.18 | 0.50 | -0.02 | -0.36 | .722 | -1.17 | 0.81 | -.02 |
| Firing them |  |  |  |  |  |  |  |  |
| (Constant) | 10.31 | 3.55 |  | 2.90 | .004 | 3.32 | 17.30 |  |
| **Female** | **2.80** | **1.43** | **0.10** | **1.96** | **.051** | **-0.01** | **5.61** | **.10** |
| Conservatism | -0.06 | 0.04 | -0.08 | -1.55 | .121 | -0.13 | 0.02 | -.08 |
| **Age** | **-1.59** | **0.54** | **-0.15** | **-2.93** | **.004** | **-2.66** | **-0.53** | **-.14** |
| Shaming them on social media | | |  |  |  |  |  |  |
| (Constant) | 24.82 | 4.87 |  | 5.10 | <.001 | 15.24 | 34.39 |  |
| Female | 2.20 | 1.96 | 0.06 | 1.12 | .262 | -1.65 | 6.05 | .05 |
| **Conservatism** | **-0.18** | **0.05** | **-0.17** | **-3.35** | **<.001** | **-0.28** | **-0.07** | **-.16** |
| **Age** | **-3.15** | **0.74** | **-0.21** | **-4.23** | **<.001** | **-4.61** | **-1.69** | **-.20** |
| Removing them from leadership positions | | | | |  |  |  |  |
| (Constant) | 36.39 | 7.12 |  | 5.11 | <.001 | 22.39 | 50.39 |  |
| **Female** | **9.05** | **2.86** | **0.16** | **3.16** | **.002** | **3.42** | **14.68** | **.15** |
| **Conservatism** | **-0.37** | **0.08** | **-0.23** | **-4.88** | **<.001** | **-0.52** | **-0.22** | **-.23** |
| **Age** | **-3.61** | **1.09** | **-0.16** | **-3.32** | **<.001** | **-5.75** | **-1.47** | **-.16** |
| Note. Bold indicates \|semipartial r\| ≥ .10. | | | |  |  |  |  |  |

**Additional Results**

**Harm Assessment**

For the question of who should determine whether social scientific conclusions pose too much risk of harm to publish or teach, the strongest support was seen for “the scholar publishing or teaching the research” (n=225), followed by “nobody—social scientific conclusions should be published and taught regardless of perceived risks” (n=190). There was weaker but still moderate support for “the members of the community that the scholar is researching/discussing” (n=141), peer scholars (n=139), university ethics committees (n=130), and journal editors (n=121). There was very little support for “university leadership” (n=22) and students (n=21).

**Main Study Qualtrics Survey**

**Start of Block: SCBEvo**

SCBEvo Please respond to the three questions below regarding this statement:

"The tendency to engage in sexually coercive behavior likely evolved because it conferred some evolutionary advantages on men who engaged in such behavior."

SCBEvo_FT How confident are you in the truth or falsity of this statement?

|  | 100% confident it is false | 100% confident it is true |
| --- | --- | --- |

|  | 0 | 10 | 20 | 30 | 40 | 50 | 60 | 70 | 80 | 90 | 100 |
| --- | --- | --- | --- | --- | --- | --- | --- | --- | --- | --- | --- |

| () | 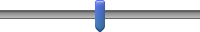 |
| --- | --- |

SCBEvo_Rel If the topic came up in a professional setting--for example, at a conference--how reluctant would you feel about sharing your beliefs on this topic openly?

|  | Not at all reluctant | Extremely reluctant |
| --- | --- | --- |

|  | 0 | 10 | 20 | 30 | 40 | 50 | 60 | 70 | 80 | 90 | 100 |
| --- | --- | --- | --- | --- | --- | --- | --- | --- | --- | --- | --- |

| () | 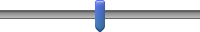 |
| --- | --- |

SBCEvo_Disc Should scholars be discouraged from testing the veracity of this statement?

|  | No discouragement | Very strong discouragement |
| --- | --- | --- |

|  | 0 | 10 | 20 | 30 | 40 | 50 | 60 | 70 | 80 | 90 | 100 |
| --- | --- | --- | --- | --- | --- | --- | --- | --- | --- | --- | --- |

| () | 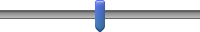 |
| --- | --- |

**End of Block: SCBEvo**

**Start of Block: STEM**

STEM Please respond to the three questions below regarding this statement:


"Gender biases are not the most important drivers of the under-representation of women in STEM fields."

STEM_FT How confident are you in the truth or falsity of this statement?

|  | 100% confident it is false | 100% confident it is true |
| --- | --- | --- |

|  | 0 | 10 | 20 | 30 | 40 | 50 | 60 | 70 | 80 | 90 | 100 |
| --- | --- | --- | --- | --- | --- | --- | --- | --- | --- | --- | --- |

| () | 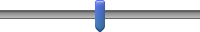 |
| --- | --- |

STEM_Rel If the topic came up in a professional setting--for example, at a conference--how reluctant would you feel about sharing your beliefs on this topic openly?

|  | Not at all reluctant | Extremely reluctant |
| --- | --- | --- |

|  | 0 | 10 | 20 | 30 | 40 | 50 | 60 | 70 | 80 | 90 | 100 |
| --- | --- | --- | --- | --- | --- | --- | --- | --- | --- | --- | --- |

| () | 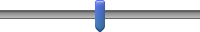 |
| --- | --- |

STEM_Disc Should scholars be discouraged from testing the veracity of this statement?

|  | No discouragement | Very strong discouragement |
| --- | --- | --- |

|  | 0 | 10 | 20 | 30 | 40 | 50 | 60 | 70 | 80 | 90 | 100 |
| --- | --- | --- | --- | --- | --- | --- | --- | --- | --- | --- | --- |

| () | 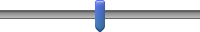 |
| --- | --- |

**End of Block: STEM**

**Start of Block: AcBlack**

AcBlack Please respond to the three questions below regarding this statement:


"Academia discriminates against Black people (e.g., in hiring, promotion, grants, invitations to participate in colloquia/symposia)."

AcBlack_FT How confident are you in the truth or falsity of this statement?

|  | 100% confident it is false | 100% confident it is true |
| --- | --- | --- |

|  | 0 | 10 | 20 | 30 | 40 | 50 | 60 | 70 | 80 | 90 | 100 |
| --- | --- | --- | --- | --- | --- | --- | --- | --- | --- | --- | --- |

| () | 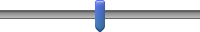 |
| --- | --- |

AcBlack_Rel If the topic came up in a professional setting--for example, at a conference--how reluctant would you feel about sharing your beliefs on this topic openly?

|  | Not at all reluctant | Extremely reluctant |
| --- | --- | --- |

|  | 0 | 10 | 20 | 30 | 40 | 50 | 60 | 70 | 80 | 90 | 100 |
| --- | --- | --- | --- | --- | --- | --- | --- | --- | --- | --- | --- |

| () | 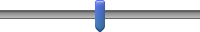 |
| --- | --- |

AcBlack_Disc Should scholars be discouraged from testing the veracity of this statement?

|  | No discouragement | Very strong discouragement |
| --- | --- | --- |

|  | 0 | 10 | 20 | 30 | 40 | 50 | 60 | 70 | 80 | 90 | 100 |
| --- | --- | --- | --- | --- | --- | --- | --- | --- | --- | --- | --- |

| () | 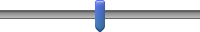 |
| --- | --- |

**End of Block: AcBlack**

**Start of Block: Binary**

Binary Please respond to the three questions below regarding this statement:


"Biological sex is binary for the vast majority of people."

Binary_FT How confident are you in the truth or falsity of this statement?

|  | 100% confident it is false | 100% confident it is true |
| --- | --- | --- |

|  | 0 | 10 | 20 | 30 | 40 | 50 | 60 | 70 | 80 | 90 | 100 |
| --- | --- | --- | --- | --- | --- | --- | --- | --- | --- | --- | --- |

| () | 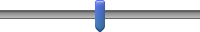 |
| --- | --- |

Binary_Rel If the topic came up in a professional setting--for example, at a conference--how reluctant would you feel about sharing your beliefs on this topic openly?

|  | Not at all reluctant | Extremely reluctant |
| --- | --- | --- |

|  | 0 | 10 | 20 | 30 | 40 | 50 | 60 | 70 | 80 | 90 | 100 |
| --- | --- | --- | --- | --- | --- | --- | --- | --- | --- | --- | --- |

| () | 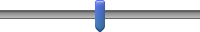 |
| --- | --- |

Binary_Disc Should scholars be discouraged from testing the veracity of this statement?

|  | No discouragement | Very strong discouragement |
| --- | --- | --- |

|  | 0 | 10 | 20 | 30 | 40 | 50 | 60 | 70 | 80 | 90 | 100 |
| --- | --- | --- | --- | --- | --- | --- | --- | --- | --- | --- | --- |

| () | 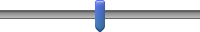 |
| --- | --- |

**End of Block: Binary**

**Start of Block: Cons**

Cons Please respond to the three questions below regarding this statement:


"The social sciences (in the United States) discriminate against conservatives (e.g., in hiring, promotion, grants, invitations to participate in colloquia/symposia)."

Cons_FT How confident are you in the truth or falsity of this statement?

|  | 100% confident it is false | 100% confident it is true |
| --- | --- | --- |

|  | 0 | 10 | 20 | 30 | 40 | 50 | 60 | 70 | 80 | 90 | 100 |
| --- | --- | --- | --- | --- | --- | --- | --- | --- | --- | --- | --- |

| () | 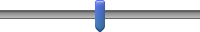 |
| --- | --- |

Cons_Rel If the topic came up in a professional setting--for example, at a conference--how reluctant would you feel about sharing your beliefs on this topic openly?

|  | Not at all reluctant | Extremely reluctant |
| --- | --- | --- |

|  | 0 | 10 | 20 | 30 | 40 | 50 | 60 | 70 | 80 | 90 | 100 |
| --- | --- | --- | --- | --- | --- | --- | --- | --- | --- | --- | --- |

| () | 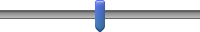 |
| --- | --- |

Cons_Disc Should scholars be discouraged from testing the veracity of this statement?

|  | No discouragement | Very strong discouragement |
| --- | --- | --- |

|  | 0 | 10 | 20 | 30 | 40 | 50 | 60 | 70 | 80 | 90 | 100 |
| --- | --- | --- | --- | --- | --- | --- | --- | --- | --- | --- | --- |

| () | 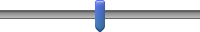 |
| --- | --- |

**End of Block: Cons**

**Start of Block: Crime**

Crime Please respond to the three questions below regarding this statement:


"Racial biases are not the most important drivers of higher crime rates among Black Americans relative to White Americans."

Crime_FT How confident are you in the truth or falsity of this statement?

|  | 100% confident it is false | 100% confident it is true |
| --- | --- | --- |

|  | 0 | 10 | 20 | 30 | 40 | 50 | 60 | 70 | 80 | 90 | 100 |
| --- | --- | --- | --- | --- | --- | --- | --- | --- | --- | --- | --- |

| () | 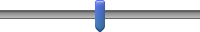 |
| --- | --- |

Crime_Rel If the topic came up in a professional setting--for example, at a conference--how reluctant would you feel about sharing your beliefs on this topic openly?

|  | Not at all reluctant | Extremely reluctant |
| --- | --- | --- |

|  | 0 | 10 | 20 | 30 | 40 | 50 | 60 | 70 | 80 | 90 | 100 |
| --- | --- | --- | --- | --- | --- | --- | --- | --- | --- | --- | --- |

| () | 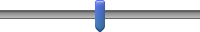 |
| --- | --- |

Crime_Disc Should scholars be discouraged from testing the veracity of this statement?

|  | No discouragement | Very strong discouragement |
| --- | --- | --- |

|  | 0 | 10 | 20 | 30 | 40 | 50 | 60 | 70 | 80 | 90 | 100 |
| --- | --- | --- | --- | --- | --- | --- | --- | --- | --- | --- | --- |

| () | 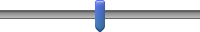 |
| --- | --- |

**End of Block: Crime**

**Start of Block: MFEvo**

MFEvo Please respond to the three questions below regarding this statement:


"Men and women have different psychological characteristics because of evolution."

MFEvo_FT How confident are you in the truth or falsity of this statement?

|  | 100% confident it is false | 100% confident it is true |
| --- | --- | --- |

|  | 0 | 10 | 20 | 30 | 40 | 50 | 60 | 70 | 80 | 90 | 100 |
| --- | --- | --- | --- | --- | --- | --- | --- | --- | --- | --- | --- |

| () | 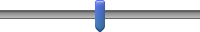 |
| --- | --- |

MFEvo_Rel If the topic came up in a professional setting--for example, at a conference--how reluctant would you feel about sharing your beliefs on this topic openly?

|  | Not at all reluctant | Extremely reluctant |
| --- | --- | --- |

|  | 0 | 10 | 20 | 30 | 40 | 50 | 60 | 70 | 80 | 90 | 100 |
| --- | --- | --- | --- | --- | --- | --- | --- | --- | --- | --- | --- |

| () | 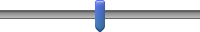 |
| --- | --- |

MFEvo_Disc Should scholars be discouraged from testing the veracity of this statement?

|  | No discouragement | Very strong discouragement |
| --- | --- | --- |

|  | 0 | 10 | 20 | 30 | 40 | 50 | 60 | 70 | 80 | 90 | 100 |
| --- | --- | --- | --- | --- | --- | --- | --- | --- | --- | --- | --- |

| () | 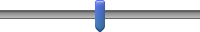 |
| --- | --- |

**End of Block: MFEvo**

**Start of Block: RaceIQ**

RaceIQ Please respond to the three questions below regarding this statement:


"Genetic differences explain non-trivial (10% or more) variance in race differences in intelligence test scores."

RaceIQ_FT How confident are you in the truth or falsity of this statement?

|  | 100% confident it is false | 100% confident it is true |
| --- | --- | --- |

|  | 0 | 10 | 20 | 30 | 40 | 50 | 60 | 70 | 80 | 90 | 100 |
| --- | --- | --- | --- | --- | --- | --- | --- | --- | --- | --- | --- |

| () | 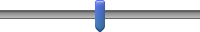 |
| --- | --- |

RaceIQ_Rel If the topic came up in a professional setting--for example, at a conference--how reluctant would you feel about sharing your beliefs on this topic openly?

|  | Not at all reluctant | Extremely reluctant |
| --- | --- | --- |

|  | 0 | 10 | 20 | 30 | 40 | 50 | 60 | 70 | 80 | 90 | 100 |
| --- | --- | --- | --- | --- | --- | --- | --- | --- | --- | --- | --- |

| () | 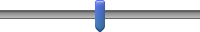 |
| --- | --- |

RaceIQ_Disc Should scholars be discouraged from testing the veracity of this statement?

|  | No discouragement | Very strong discouragement |
| --- | --- | --- |

|  | 0 | 10 | 20 | 30 | 40 | 50 | 60 | 70 | 80 | 90 | 100 |
| --- | --- | --- | --- | --- | --- | --- | --- | --- | --- | --- | --- |

| () | 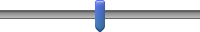 |
| --- | --- |

**End of Block: RaceIQ**

**Start of Block: TransSI**

TransSI Please respond to the three questions below regarding this statement:


"Transgender identity is sometimes the product of social influence."

TransSI_FT How confident are you in the truth or falsity of this statement?

|  | 100% confident it is false | 100% confident it is true |
| --- | --- | --- |

|  | 0 | 10 | 20 | 30 | 40 | 50 | 60 | 70 | 80 | 90 | 100 |
| --- | --- | --- | --- | --- | --- | --- | --- | --- | --- | --- | --- |

| () | 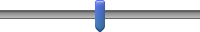 |
| --- | --- |

TransSI_Rel If the topic came up in a professional setting--for example, at a conference--how reluctant would you feel about sharing your beliefs on this topic openly?

|  | Not at all reluctant | Extremely reluctant |
| --- | --- | --- |

|  | 0 | 10 | 20 | 30 | 40 | 50 | 60 | 70 | 80 | 90 | 100 |
| --- | --- | --- | --- | --- | --- | --- | --- | --- | --- | --- | --- |

| () | 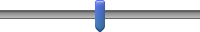 |
| --- | --- |

TransSI_Disc Should scholars be discouraged from testing the veracity of this statement?

|  | No discouragement | Very strong discouragement |
| --- | --- | --- |

|  | 0 | 10 | 20 | 30 | 40 | 50 | 60 | 70 | 80 | 90 | 100 |
| --- | --- | --- | --- | --- | --- | --- | --- | --- | --- | --- | --- |

| () | 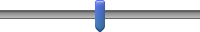 |
| --- | --- |

**End of Block: TransSI**

**Start of Block: Divers**

Divers Please respond to the three questions below regarding this statement:


"Demographic diversity (race, gender) in the workplace often leads to worse performance."

Divers_FT How confident are you in the truth or falsity of this statement?

|  | 100% confident it is false | 100% confident it is true |
| --- | --- | --- |

|  | 0 | 10 | 20 | 30 | 40 | 50 | 60 | 70 | 80 | 90 | 100 |
| --- | --- | --- | --- | --- | --- | --- | --- | --- | --- | --- | --- |

| () | 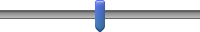 |
| --- | --- |

Divers_Rel If the topic came up in a professional setting--for example, at a conference--how reluctant would you feel about sharing your beliefs on this topic openly?

|  | Not at all reluctant | Extremely reluctant |
| --- | --- | --- |

|  | 0 | 10 | 20 | 30 | 40 | 50 | 60 | 70 | 80 | 90 | 100 |
| --- | --- | --- | --- | --- | --- | --- | --- | --- | --- | --- | --- |

| () | 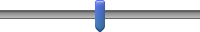 |
| --- | --- |

Divers_DIsc Should scholars be discouraged from testing the veracity of this statement?

|  | No discouragement | Very strong discouragement |
| --- | --- | --- |

|  | 0 | 10 | 20 | 30 | 40 | 50 | 60 | 70 | 80 | 90 | 100 |
| --- | --- | --- | --- | --- | --- | --- | --- | --- | --- | --- | --- |

| () | 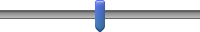 |
| --- | --- |

**End of Block: Divers**

**Start of Block: Round2 Questions**

Transition Thank you. Now you will move on to broader questions.

| Page Break |  |
| --- | --- |

OpenRisks Taking into consideration all of your views on these topics, if you were to share these views openly, how at risk would you feel for the following consequences?

|  | No risk at all | Very high risk |
| --- | --- | --- |

|  | 0 | 10 | 20 | 30 | 40 | 50 | 60 | 70 | 80 | 90 | 100 |
| --- | --- | --- | --- | --- | --- | --- | --- | --- | --- | --- | --- |

| Being ostracized by some peers () | 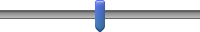 |
| --- | --- |
| Career damaging biases against me (e.g., in publishing, promotion, awards, grants, talk invites) () | 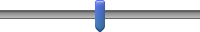 |
| Being stigmatized or labeled pejorative terms () | 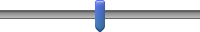 |
| Disciplinary actions (e.g, losing classes, losing leadership roles, formal reprimand) () | 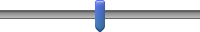 |
| Guilt-by-association harm to my students and colleagues () | 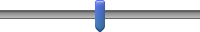 |
| Being fired () | 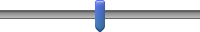 |
| Being attacked on social media () | 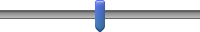 |
| Student boycotts () | 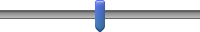 |
| Threats of physical violence () | 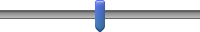 |

| Page Break |  |
| --- | --- |

Taboo Are there conclusions in the social sciences that have some empirical support but are nonetheless taboo (in that mentioning the supportive evidence would lead to formal or informal punishment)?

|  | Definitely not | Yes, definitely |
| --- | --- | --- |

|  | 0 | 10 | 20 | 30 | 40 | 50 | 60 | 70 | 80 | 90 | 100 |
| --- | --- | --- | --- | --- | --- | --- | --- | --- | --- | --- | --- |

| () | 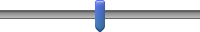 |
| --- | --- |

Example If yes, please list an example.

________________________________________________________________

| Page Break |  |
| --- | --- |

Retract Which of the following are legitimate reasons to retract a paper?”

|  | Never legitimate | Always legitimate |
| --- | --- | --- |

|  | 0 | 10 | 20 | 30 | 40 | 50 | 60 | 70 | 80 | 90 | 100 |
| --- | --- | --- | --- | --- | --- | --- | --- | --- | --- | --- | --- |

| Data fraud (i.e., making up or altering data) () | 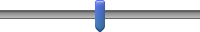 |
| --- | --- |
| Analytic errors that alter primary conclusion () | 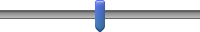 |
| Numerous failures to replicate () | 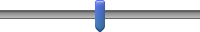 |
| Compelling evidence of p-hacking (e.g., p-curve) () | 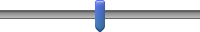 |
| Failure to obtain ethics approval () | 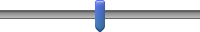 |
| Moral concerns that the conclusions could harm vulnerable groups () | 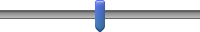 |
| The risk of extremists misconstruing and weaponizing the results () | 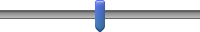 |

| Page Break |  |
| --- | --- |

Fire Which of the following are legitimate reasons to fire a scholar?”

|  | Never legitimate | Always legitimate |
| --- | --- | --- |

|  | 0 | 10 | 20 | 30 | 40 | 50 | 60 | 70 | 80 | 90 | 100 |
| --- | --- | --- | --- | --- | --- | --- | --- | --- | --- | --- | --- |

| Data fraud (i.e., making up or altering data) () | 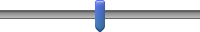 |
| --- | --- |
| Numerous failures to replicate their findings () | 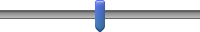 |
| Compelling evidence of p-hacking in more than one paper () | 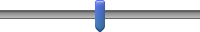 |
| Engaging in sexual behavior with their own graduate or undergraduate students () | 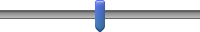 |
| Moral concerns about the implications of their research conclusions () | 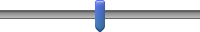 |
| Their research has become popular with extremist groups () | 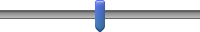 |

| Page Break |  |
| --- | --- |

Petitioners How much admiration vs. contempt do you hold toward peers who start petitions or social media campaigns to retract papers for the following reasons?

|  | Maximum contempt | Maximum admiration |
| --- | --- | --- |

|  | 0 | 10 | 20 | 30 | 40 | 50 | 60 | 70 | 80 | 90 | 100 |
| --- | --- | --- | --- | --- | --- | --- | --- | --- | --- | --- | --- |

| Data fraud (i.e., making up or altering data) () | 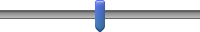 |
| --- | --- |
| Research error (e.g., mistake in analysis) () | 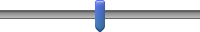 |
| Moral concerns about the conclusions (e.g., findings reinforce negative stereotypes) () | 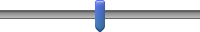 |

| Page Break |  |
| --- | --- |

Free Should scholars be completely free to pursue research questions without fear of institutional punishment (e.g., from their university, journals, or professional societies) for their conclusions?

- No (1)
- It's Complicated (2)
- Yes (3)

| Page Break |  |
| --- | --- |

SEvTruth If pursuit of truth and social equity goals appear to come into conflict, which should scientists prioritize?

- Social Equity (1)
- It's Complicated (2)
- Truth (3)

| Page Break |  |
| --- | --- |

RiskPolice Who should determine whether social scientific conclusions pose too much risk of harm to publish or teach? (tick all that apply)

- University leadership (e.g., Presidents, Provosts, Deans, Chairs) (1)
- University ethics committees (2)
- Journal editors (3)
- Peer scholars (e.g., concerns expressed in petitions, social media campaigns) (10)
- The members of the community that the scholar is researching/discussing (11)
- Students (12)
- The scholar publishing or teaching the research (13)
- Nobody--social scientific conclusions should be published and taught regardless of perceived harm risks (14)

| Page Break |  |
| --- | --- |

HarmLikelihood How certain should it be that a social scientific finding is going to cause harm before it should be suppressed?

- The harm should seem possible (1)
- The harm should seem likely (2)
- There should be suggestive evidence it would cause harm (3)
- There should be clear evidence it would cause harm (4)
- There should be evidence that the only way to avoid the harm is to suppress the research (5)
- We should never suppress social scientific findings (6)

| Page Break |  |
| --- | --- |

TabooPunishment In our earlier interviews with psychology professors, we discovered the most taboo conclusions in the social sciences tend to involve genetic or evolutionary explanations for group differences in socially valued outcomes (e.g., education and career outcomes, SES, criminal justice involvement), and particularly in domains where women underperform relative to men or where Black people underperform relative to White people. Now imagine a scholar who forwarded a genetic or evolutionary explanation for gender or racial differences in socially valued outcomes in their research. Which of the following actions would you support against him or her?

|  | Would not support at all | Would strongly support |
| --- | --- | --- |

|  | 0 | 10 | 20 | 30 | 40 | 50 | 60 | 70 | 80 | 90 | 100 |
| --- | --- | --- | --- | --- | --- | --- | --- | --- | --- | --- | --- |

| Normal scientific criticism (e.g., commentaries about perceived errors) () | 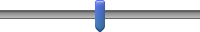 |
| --- | --- |
| Socially ostracizing them () | 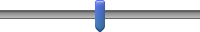 |
| Publicly labeling them pejorative terms (e.g., bigot, racist, sexist) () | 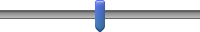 |
| Disinviting them from talks () | 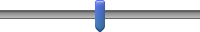 |
| Refusing to publish their work regardless of its merits () | 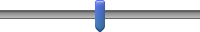 |
| Not hiring or promoting them even if they meet typical standards () | 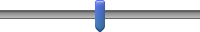 |
| Stigmatizing their graduate students and co-authors () | 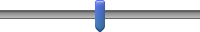 |
| Firing them () | 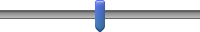 |
| Shaming them on social media () | 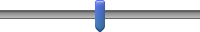 |
| Removing them from leadership positions () | 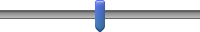 |

| Page Break |  |
| --- | --- |

OpenResponse There are only demographic questions left. Is there anything you would like to tell us?

________________________________________________________________

________________________________________________________________

________________________________________________________________

________________________________________________________________

________________________________________________________________

**End of Block: Round2 Questions**

**Start of Block: Demogs**

Position Which most closely describes your position?

- Postdoc (1)
- Teaching Faculty (2)
- Assistant Professor (3)
- Associate Professor (4)
- Professor (5)
- Professor Emeritus (6)
- Other Research Faculty (7)
- Adjunct (8)
- None of these (9)

Subdiscipline Which of the following psychology subdisciplines are closest to yours? (multiple responses allowed)

- Behavioral Genetics (1)
- Biological (2)
- Clinical (4)
- Cognitive (5)
- Counseling (6)
- Developmental (7)
- Educational (8)
- Health (9)
- Industrial/Organizational (10)
- Neuroscience (11)
- Social and Personality (12)
- Other (13)
- I am not a psychologist (14)

Gender Gender

- Male (1)
- Female (2)
- Nonbinary (3)
- Do not wish to disclose (4)

| 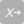 |
| --- |

Race Please choose whichever race or ethnicity that you identify with (you may choose more than one):

- Asian (1)
- Black (2)
- Hispanic (3)
- Middle Eastern (4)
- Native American or Indigenous (5)
- Non-Hispanic White (7)
- Other (specify) (6) ________________________________________________

Age Age group

- 18-25 (1)
- 26-35 (2)
- 36-45 (3)
- 46-55 (4)
- 56-65 (5)
- 66-75 (6)
- 76+ (7)

Ideo How would you rate your overall political ideology?

|  | Extremely liberal | Moderate/Centrist | Extremely conservative |
| --- | --- | --- | --- |

|  | 0 | 10 | 20 | 30 | 40 | 50 | 60 | 70 | 80 | 90 | 100 |
| --- | --- | --- | --- | --- | --- | --- | --- | --- | --- | --- | --- |

| () | 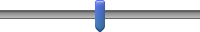 |
| --- | --- |

SocIdeo How would you rate your political ideology on social issues?

|  | Extremely liberal | Moderate/Centrist | Extremely conservative |
| --- | --- | --- | --- |

|  | 0 | 10 | 20 | 30 | 40 | 50 | 60 | 70 | 80 | 90 | 100 |
| --- | --- | --- | --- | --- | --- | --- | --- | --- | --- | --- | --- |

| () | 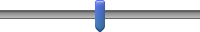 |
| --- | --- |

EconIdeo How would you rate your political ideology on economic issues?

|  | Extremely liberal | Moderate/Centrist | Extremely conservative |
| --- | --- | --- | --- |

|  | 0 | 10 | 20 | 30 | 40 | 50 | 60 | 70 | 80 | 90 | 100 |
| --- | --- | --- | --- | --- | --- | --- | --- | --- | --- | --- | --- |

| () | 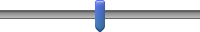 |
| --- | --- |

1. One participant said it was “good if you want to start an empire and take control like China and Russia but bad if you want a classic Western education.” This was assumed to be a joke and coded as “bad” rather than “neither/both.” [↑](#footnote-ref-1)
2. Note many people mentioned multiple topics and conclusions, so total responses are greater than the total number of participants. For follow-up questions, they were asked to choose one, but some participants continued to respond to multiple. [↑](#footnote-ref-2)
3. Three participants broke the most taboo conclusions into two or three subconclusions and gave different estimates for each. For these participants, only their first response was included in the average and follow-up analyses. [↑](#footnote-ref-3)
4. Once again, if participants provided multiple answers, only their first was included. [↑](#footnote-ref-4)
